# Effect of Gram Stain–Guided Initial Antibiotic Therapy on Clinical Response in Patients With Ventilator-Associated Pneumonia: The GRACE-VAP Randomized Clinical Trial

**DOI:** 10.1001/jamanetworkopen.2022.6136

**Published:** 2022-04-08

**Authors:** Jumpei Yoshimura, Kazuma Yamakawa, Yoshinori Ohta, Kensuke Nakamura, Hideki Hashimoto, Masahiro Kawada, Hiroki Takahashi, Takeshi Yamagiwa, Akira Kodate, Kyohei Miyamoto, Satoshi Fujimi, Takeshi Morimoto

**Affiliations:** 1 Division of Trauma and Surgical Critical Care, Osaka General Medical Center, Sumiyoshi, Osaka, Japan; 2 Department of Traumatology and Acute Critical Medicine, Osaka University Graduate School of Medicine, Suita, Japan; 3 Department of Emergency Medicine, Osaka Medical and Pharmaceutical University, Takatsuki, Osaka, Japan; 4 Education and Training Center for Students and Professionals in Healthcare, Hyogo College of Medicine, Nishinomiya, Hyogo, Japan; 5 Department of Emergency and Critical Care Medicine, Hitachi General Hospital, Hitachi, Ibaraki, Japan; 6 Department of Emergency and Critical Care Medicine, Kansai Medical University Medical Center, Moriguchi, Osaka, Japan; 7 Department of Emergency and Critical Care Medicine, Kansai Medical University Hospital, Hirakata, Osaka, Japan; 8 Department of Emergency Medicine, Ebina General Hospital, Ebina, Kanagawa, Japan; 9 Department of Emergency and Critical Care Medicine, Sapporo City General Hospital, Chuoh, Sapporo, Hokkaido, Japan; 10 Department of Emergency and Critical Care Medicine, Wakayama Medical University, Wakayama, Japan; 11 Department of Clinical Epidemiology, Hyogo College of Medicine, Nishinomiya, Hyogo, Japan

## Abstract

**Importance:**

Gram staining should provide immediate information for detecting causative pathogens. However, the effect of Gram staining on restricting the initial antibiotic choice has not been investigated in intensive care units (ICUs).

**Objective:**

To compare the clinical response to Gram stain–guided restrictive antibiotic therapy vs guideline-based broad-spectrum antibiotic treatment in patients with ventilator-associated pneumonia (VAP).

**Design, Setting, and Participants:**

This multicenter, open-label, noninferiority randomized clinical trial (Gram Stain-Guided Antibiotics Choice for VAP) was conducted in the ICUs of 12 tertiary referral hospitals in Japan from April 1, 2018, through May 31, 2020. Patients aged 15 years or older with a VAP diagnosis and a modified Clinical Pulmonary Infection Score of 5 or higher were included. The primary analysis was based on the per-protocol analysis population.

**Interventions:**

Patients were randomized to Gram stain–guided antibiotic therapy or guideline-based antibiotic therapy (based on the 2016 Infectious Disease Society of America and American Thoracic Society clinical practice guidelines for VAP).

**Main Outcomes and Measures:**

The primary outcome was the clinical response rate; clinical response was defined as completion of antibiotic therapy within 14 days, improvement or lack of progression of baseline radiographic findings, resolution of signs and symptoms of pneumonia, and lack of antibiotic agent readministration, with a noninferiority margin of 20%. Secondary outcomes were the proportions of antipseudomonal agents and anti–methicillin-resistant *Staphylococcus aureus* (MRSA) agents as initial antibiotic therapies; 28-day mortality, ICU-free days, ventilator-free days; and adverse events.

**Results:**

In total, 206 patients (median [IQR] age, 69 [54-78] years; 141 men [68.4%]) were randomized to the Gram stain–guided group (n = 103) or guideline-based group (n = 103). Clinical response occurred in 79 patients (76.7%) in the Gram stain–guided group and 74 patients (71.8%) in the guideline-based group (risk difference, 0.05; 95% CI, –0.07 to 0.17; *P* < .001 for noninferiority). Reduced use of antipseudomonal agents (30.1%; 95% CI, 21.5%-39.9%; *P* < .001) and anti-MRSA agents (38.8%; 95% CI, 29.4%-48.9%; *P* < .001) was observed in the Gram stain–guided group vs guideline-based group. The 28-day cumulative incidence of mortality was 13.6% (n = 14) in the Gram stain–guided group vs 17.5% (n = 18) in the guideline-based group (*P* = .39). Escalation of antibiotics according to culture results was performed in 7 patients (6.8%) in the Gram stain–guided group and 1 patient (1.0%) in the guideline-based group (*P* = .03). There were no significant differences between the groups in ICU-free days, ventilator-free days, and adverse events.

**Conclusions and Relevance:**

Results of this trial showed that Gram stain–guided treatment was noninferior to guideline-based treatment and significantly reduced the use of broad-spectrum antibiotics in patients with VAP. Gram staining can potentially ameliorate the multidrug-resistant organisms in the critical care setting.

**Trial Registration:**

ClinicalTrials.gov Identifier: NCT03506113

## Key Points

**Question:**

Does Gram stain–guided antibiotic therapy restrict the administration of broad-spectrum antibiotic agents for ventilator-associated pneumonia without detrimental effects on patient outcomes?

**Findings:**

In this randomized clinical trial that included 206 patients with ventilator-associated pneumonia in the intensive care unit, the clinical response to Gram stain–guided antibiotic therapy was noninferior to that of guideline-based antibiotic therapy (76.7% vs 71.8%). Gram stain–guided antibiotic therapy reduced the use of antipseudomonal agents and anti–methicillin-resistant *Staphylococcus aureus* agents.

**Meaning:**

The findings of this trial suggest that Gram staining can be used in the critical care setting to ameliorate the spread of multidrug-resistant pathogens.

## Teaser

This randomized clinical trial compares the clinical response to Gram stain–guided vs guideline-based antibiotic therapy in patients with ventilator-associated pneumonia.

## Introduction

The rapid spread of multidrug-resistant (MDR) organisms in intensive care units (ICUs) is recognized as a worldwide health threat,^1,2^ but development of new pharmaceutical agents is lagging, leaving a decreasing stock of effective antibiotics against these organisms.^3,4^ Therefore, a global action plan to address antimicrobial resistance was devised by the World Health Organization to optimize the use of antibiotic agents.^5^

Patients in the ICU are among the largest consumers of antibiotics in the hospital because they are at an increased risk for infection owing to underlying conditions, impaired immunity, and exposure to multiple invasive devices.^6^ Several studies found that 50% to 70% of patients in the ICU had infections and were given antibiotics during their ICU stay.^7,8,9^ Among the threats of infection in ICUs, ventilator-associated pneumonia (VAP) is an important complication that necessitates antibiotics in approximately 10% of patients undergoing mechanical ventilation and that results in an estimated attributable mortality of 15% to 50%.^10,11,12^ The 2016 clinical practice guidelines for VAP published by the Infectious Disease Society of America and American Thoracic Society recommend coverage for both methicillin-resistant *Staphylococcus aureus* (MRSA) and *Pseudomonas aeruginosa* as an empirical treatment.^13^ However, overuse of broad-spectrum antibiotic agents because of these guidelines could be associated with the accelerated emergence of antimicrobial-resistant organisms.^14^ Thus, establishing methods to safely reduce overuse of broad-spectrum antibiotic agents for VAP is a pressing challenge.

Gram staining of respiratory samples, an inexpensive, simple examination that is used worldwide, including in lower-income countries, may guide the appropriate use of initial antibiotic therapy. Several studies evaluated the accuracy of Gram staining in detecting causative pathogens, but their results were conflicting.^15,16,17,18,19^ Furthermore, the question of whether Gram staining results are accurate enough to safely restrict the use of broad-spectrum antibiotics remains controversial. Therefore, we conducted the Gram Stain-Guided Antibiotics Choice for VAP (GRACE-VAP) trial to compare the clinical response to Gram stain–guided restrictive antibiotic treatment vs guideline-based broad-spectrum antibiotic treatment in patients with VAP.

## Methods

### Design, Setting, and Patients

The GRACE-VAP trial was a multicenter, open-label, noninferiority randomized clinical trial with blinded end point assessment that was conducted from April 1, 2018, through May 31, 2020. The trial protocol (provided in Supplement 1 and a protocol paper^20^) was concordant with the Declaration of Helsinki^21^ and the Ethical Guidelines for Medical and Health Research Involving Human Subjects in Japan and was approved by the institutional review boards of Osaka General Medical Center and each of the participating institutions. Written informed consent was obtained from all participants or their representatives. We followed the Consolidated Standards of Reporting Trials (CONSORT) reporting guideline.

The trial recruited patients from ICUs of 12 tertiary referral hospitals in Japan (Chukyo Hospital, Nagoya, Aichi; Ebina General Hospital, Ebina, Kanagawa; Hitachi General Hospital, Hitachi, Ibaraki; Kansai Medical University Hospital, Hirakata, Osaka; Kansai Medical University Medical Center, Moriguchi, Osaka; Nagasaki University Hospital, Nagasaki; Osaka General Medical Center, Osaka; Saga University Hospital, Saga; Sapporo City General Hospital, Sapporo, Hokkaido; Tajima Emergency and Critical Care Medical Center, Toyooka, Hyogo; University of the Ryukyus Hospital, Nakagami-gun, Okinawa; and Wakayama Medical University Hospital, Wakayama) (eTable 1 in Supplement 2). An independent data center and Data and Safety Monitoring Board provided trial oversight (eTable 2 in Supplement 2).

Patients were eligible if they were 15 years or older; had undergone mechanical ventilation for at least 48 hours; and had been diagnosed with VAP, defined as a modified Clinical Pulmonary Infection Score (mCPIS) of 5 or higher (score range: 0-10, with higher scores indicating greater likelihood of VAP).^22^ The exclusion criteria were known allergy to study medications, pregnancy, discharge from ICU, heart failure or atelectasis diagnosis, receipt of antibiotics for more than 24 hours after meeting the inclusion criteria, withdrawal or withholding of life support, or the discretion of the site investigator. We also excluded patients who were diagnosed with COVID-19 infection as an ex-post criterion when COVID-19 became pandemic in February 2020.

Data on race and ethnicity were not collected. It was presumed that only Japanese citizens would participate in the trial.

### Randomization and Blinding

**Figure 1.**
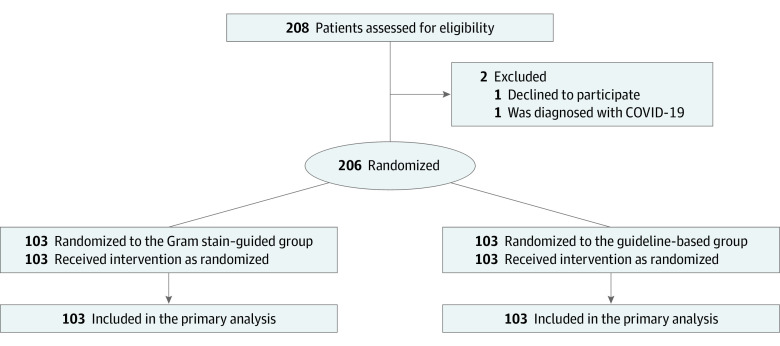
Study Flow Diagram

Patients were randomized to receive either Gram stain–guided antibiotic treatment or guideline-based standard treatment (Figure 1). Randomization was performed with a stochastic minimization procedure and was stratified by study center; presence of chronic obstructive pulmonary disease, traumatic brain injury (TBI), and postcardiopulmonary arrest syndrome; and previous antibiotic therapy during the hospitalization. We used an electronic data capture system to conduct randomization and data collection. Site investigators were not blinded to the group randomization.

### Interventions

Patients in the Gram stain–guided group received antibiotics according to the results of Gram staining of endotracheal aspirate (eFigure 1 in Supplement 2).^15,16,23^ Gram staining was performed in the laboratory in each ICU or the microbiology laboratory in each institution just after respiratory samples for sputum culture were collected. Bronchoalveolar lavage was not performed to obtain respiratory samples. Gram stain was performed by the Favor method, in which heat-fixed smears on slides were flooded with 0.2% Victoria blue for 30 seconds and washed with tap water, smears were decolorized with 2% picric acid ethanol, and cells were counterstained with 0.004% fuchsin for 30 seconds and then washed with tap water.^15^ Respiratory sample quality was assessed by Miller and Jones classification and Geckler classification. We categorized the Gram stain results as gram-positive cocci (GPC) chains, GPC clusters, gram-positive bacilli, gram-negative rods (GNR), or a combination thereof.^15,16^ A nonpseudomonal β-lactam antibiotic was administered when Gram stain results showed only GPC chains and/or gram-positive bacilli. An anti-MRSA agent was administered when Gram stain results showed GPC clusters without GNR. An antipseudomonal agent was administered when Gram stain results showed GNR without GPC clusters. The combination of an antipseudomonal agent and anti-MRSA agent was administered when Gram stain results showed both GPC clusters and GNR. The most broad-spectrum antibiotic agent among the categories was administered when Gram stain results showed 2 or more categories. We administered the combination of an antipseudomonal agent and an anti-MRSA agent when the Gram stain did not show any microorganisms.

Patients in the guideline-based group received the combination of an antipseudomonal agent and an anti-MRSA agent according to the 2016 VAP guidelines.^13^ We did not administer 2 antipseudomonal agents from different classes as initial antibiotic therapy in either group because *P. aeruginosa* remained susceptible to antipseudomonal β-lactam (80%-90%), and the frequency of MDR GNR was low (<10%) in all participating institutions (eTable 3 in Supplement 2).

In both groups, escalation of the initial treatment selection was performed to cover all pathogens that were isolated from respiratory samples before the onset of VAP.^16^ We selected specific antibiotic agents according to previously recorded antimicrobial resistance patterns in each ICU (eTable 3 in Supplement 2).

The study medication could be de-escalated or escalated to a definitive treatment level according to the pathogens that were isolated from respiratory samples. The site investigator adjusted dose regimens according to individual patient status. Study medications were continued for at least 7 days and discontinued at the discretion of the site investigator.

### Outcomes

We performed clinical assessments at baseline and daily throughout the study treatment, at the end of therapy, and at the follow-up or test-of-cure visit (7 days after the end of therapy). The primary outcome was the clinical response rate at the follow-up or test-of-cure visit. Clinical response was defined as meeting the following 4 outcomes: completion of antibiotic therapy within 14 days, improvement or lack of progression of baseline radiographic findings at the end of therapy, resolution of signs and symptoms of pneumonia (exacerbation of fever, increased oxygen dose, and cough) at the follow-up or test-of-cure visit, and lack of antibiotic agent readministration owing to pneumonia by the follow-up or test-of-cure visit.

The secondary outcomes were 28-day mortality; 28-day ICU-free days; 28-day ventilator-free days; proportions of antipseudomonal agents and anti-MRSA agents as initial antibiotic therapies; coverage rate of initial antibiotic therapies; de-escalation rate; antibiotic therapy days; and adverse events, including kidney impairment, thrombocytopenia, diarrhea, *Clostridioides difficile* infection, skin rash, and seizure. Kidney impairment was defined as both an increase in creatinine level to at least 1.5 times over baseline within the follow-up or test-of-cure period, with the worst creatinine level being 1.0 mg/dL or higher. We considered the therapy appropriate when all pathogens that were isolated with at least 1 semiquantitative growth from respiratory samples were covered by the selected antibiotic agents.

The site investigators collected information that was relevant to the outcomes. Each member of the event adjudication committee, who was blinded to the group randomization, independently judged the primary and secondary outcomes according to the individual data. When any disagreements occurred among the committee members, a consensus was reached by discussion.

### Statistical Analysis

The primary efficacy analysis assessed the noninferiority of the clinical response in the Gram stain–guided group compared with the guideline-based group. Details of the statistical analyses were described previously,^20^ and the statistical analysis plan was fixed before the enrollment of the final patient.

Sample size was based on an assumed clinical response rate of 67.8% to both the guideline-based treatment and the Gram stain–guided treatment, as determined in a previous study,^16^ and a 20% noninferiority margin. This trial required 86 patients per group to achieve 80% power with a 1-sided α = 2.5%. Assuming a 10% rate of nonevaluable patients, we decided to enroll 100 patients per group.

Categorical variables were expressed as frequencies with percentages, and continuous variables were expressed as medians with IQRs. Because the noninferiority of the primary outcome was assessed, the primary analysis was performed in the per-protocol analysis population, which consisted of all randomized patients who were not lost to follow-up and had no major protocol deviations. We verified the noninferiority of the primary outcome using the normal theory test for binomial proportions. We performed a sensitivity analysis for primary efficacy analysis in the patients with an mCPIS of 5 or higher because some patients who were considered eligible at randomization were found to have an mCPIS of 4 after data monitoring.

Secondary outcomes were analyzed under a superiority assumption, which was based on the intention-to-treat principle. The cumulative incidence of mortality at 28 days was estimated with the Kaplan-Meier method, and the differences between groups were assessed by log-rank test. The hazard ratios and 95% CIs for mortality in the Gram stain–guided group vs the guideline-based group were estimated with a Cox proportional hazards regression model. Other secondary outcomes were compared using the χ^2^ test for categorical variables and Wilcoxon rank sum test for continuous variables.

Predefined subgroup analyses for the primary outcome were conducted with the same method used in the primary efficacy analysis. The subgroup factors, which affected a priori the bacterial flora of a patient’s oral cavity and airway or were related to patient severity, included the presence of previous antibiotic therapy during hospitalization, length of ICU stay before randomization (≥5 or <5 days), comorbidity of chronic obstructive pulmonary disease, TBI, postcardiopulmonary arrest syndrome, presence of sepsis, development of acute kidney injury, and Acute Physiology and Chronic Health Evaluation II score (≥20 or <20; score range: 0-71, with higher scores indicating a higher risk of death). Because few patients had septic shock in the trial, we changed the predefined subgroup from septic shock to sepsis.

All *P* values were 2-sided, and *P* < .05 was considered to be significant except in the noninferiority test for clinical response, for which a 1-sided *P* < .025 was considered to be significant. All statistical analyses were performed with Stata, version 16.1 (StataCorp LLC) and SAS, version 9.4 (SAS Institute Inc).

## Results

Of the 208 patients with VAP who were assessed for eligibility, 2 were excluded (1 for declining participation, and 1 for a COVID-19 diagnosis). As a result, we randomized 206 patients, 103 of whom were randomized to the Gram stain–guided group and 103 to the guideline-based group (Figure 1). The sample had a median (IQR) age of 69 (54-78) years and was composed of 141 men (68.4%) and 65 women (31.6%). The median (IQR) hospital stay from ICU admission to randomization was 4 (4-6) days. Demographic data, reason for ICU admission, morbidities, and severity of illness were well balanced between the 2 groups (Table 1). *Staphylococcus aureus* (n = 103 [50.0%]) was the most frequently isolated bacteria from endotracheal aspirate, followed by *Klebsiella* spp (34 [16.5%]) and *Haemophilus influenza* (20 [9.7%]) (eTable 4 in Supplement 2). Summaries of the Gram staining results and selected antibiotic agents are shown in eTables 5 and 6 in Supplement 2.

**Table 1.** Baseline Characteristics of Patients^a^

Characteristic	No. (%)	
	Gram stain–guided group (n = 103)	Guideline-based group (n = 103)
Age, median (IQR), y	69 (54-78)	69 (54-78)
Female sex	37 (35.9)	28 (27.2)
Male sex	66 (64.1)	75 (72.8)
Diagnosis on ICU admission		
Trauma	28 (27.2)	27 (26.2)
Postcardiopulmonary arrest syndrome	25 (24.3)	24 (23.3)
Stroke	11 (10.7)	10 (9.7)
Other	39 (37.9)	42 (40.8)
mCPIS, median (IQR)^b^		
Overall	6 (5-7)	6 (5-7)
Temperature	1 (0-2)	1 (0-2)
Leukocytes	0 (0-1)	0 (0-2)
Pao_2_/Fio_2_	0 (0-2)	0 (0-2)
Chest radiograph	2 (2-2)	2 (2-2)
Tracheal secretions	2 (2-2)	2 (2-2)
Comorbidities		
Diabetes	27 (26.2)	26 (25.2)
Chronic		
Heart failure	22 (21.4)	16 (15.5)
Respiratory disorder	6 (5.8)	6 (5.8)
Hemodialysis	5 (4.9)	5 (4.9)
Liver cirrhosis	4 (3.9)	1 (1.0)
Immunocompromised	3 (2.9)	4 (3.9)
Previous antibiotic therapy	30 (29.1)	27 (26.2)
Length of ICU stay before randomization, d		
≥5	53 (51.5)	49 (47.6)
<5	50 (48.5)	54 (52.4)
Sepsis	36 (35.0)	33 (32.0)
Septic shock	6 (5.8)	3 (2.9)
Acute kidney injury^c^	20 (19.4)	13 (12.6)
APACHE II score, median (IQR)^d^	18 (14-24)	19 (15-23)
SOFA score, median, (IQR)^e^	7 (5-9)	5 (7-9)

Abbreviations: APACHE, Acute Physiology and Chronic Health Evaluation; ICU, intensive care unit; Fio_2_, fraction of inspired oxygen; mCPIS, modified Clinical Pulmonary Infection Score; Pao_2_, partial pressure of oxygen; SOFA, Sequential Organ Failure Assessment.

^a^ Percentages may not total to 100 because of rounding.

^b^ The mCPIS ranges from 0 to 10, with higher scores indicating greater likelihood of VAP.

^c^ Acute kidney injury was diagnosed according to the Kidney Disease: Improving Global Outcomes definition.

^d^ APACHE II scores range from 0 to 71, with higher scores indicating a higher risk of death.

^e^ SOFA scores range from 0 to 24 (from 0 to 4 for each of the 6 organ systems), with higher scores indicating more severe organ dysfunction.

### Outcomes

Per-protocol analysis showed that, at the follow-up or test-of-cure visit, 79 patients (76.7%) in the Gram stain–guided group had a clinical response compared with 74 patients (71.8%) in the guideline-based group (risk difference, 0.05; 95% CI, –0.07 to 0.17; *P* < .001 for noninferiority) (Table 2). Sensitivity analysis after excluding 12 patients with an mCPIS of 4 (5 in the Gram stain–guided group and 7 in the guideline-based group) yielded results that were similar to that of the primary analysis (risk difference, 0.06; 95% CI, –0.07 to 0.18; *P* < .001 for noninferiority). Clinical response rates at the participating institutions are shown in eTable 7 in Supplement 2.

**Table 2.** Primary and Secondary Outcomes

Outcome	No. (%)		*P* value
	Gram stain–guided group (n = 103)	Guideline-based group (n = 103)	
Primary outcome			
Clinical response rate	79 (76.7)	74 (71.8)	<.001^a^
Completion of antibiotic therapy within 14 d^b^	98 (95.1)	94 (91.3)	NA
Improvement or lack of progression of baseline radiographic findings^b^	85 (82.5)	78 (75.7)	NA
Resolution of signs and symptoms of pneumonia^b^	87 (84.5)	85 (82.5)	NA
Lack of antibiotic agent readministration^b^	85 (82.5)	85 (82.5)	NA
Secondary outcomes			
28-d mortality	14 (13.6)	18 (17.5)	.44
28-d ventilator-free days, median (IQR)	22 (15-24)	22 (18-25)	.21
28-d ICU-free days, median (IQR)	19 (15-22)	20 (16-23)	.42
Administration of antibiotic therapy			
Antipseudomonal agents	72 (69.9)	103 (100)	<.001
Anti-MRSA agents	63 (61.2)	103 (100)	<.001
Coverage rate of initial antibiotic therapy	89 (86.4)	95 (92.2)	.18
Escalation^b^	7 (6.8)	1 (1.0)	.03
De-escalation	67 (65.0)	79 (76.7)	.07
Antibiotic therapy days until de-escalation, median (IQR)	3 (2-4)	3 (2-4)	.22
Antibiotic therapy days, median (IQR)	8 (7-11)	8 (7-11)	.09

Abbreviations: ICU, intensive care unit; MRSA, methicillin-resistant *Staphylococcus aureus*.

^a^ The noninferiority of the primary outcome was analyzed using the normal theory test for binomial proportions.

^b^ Added post hoc.

The cumulative incidence of mortality at 28 days was 13.6% (n = 14) in the Gram stain–guided group and 17.5% (n = 18) in the guideline-based group (*P* = .39) (eFigure 2 in Supplement 2). The hazard ratio of the Gram stain–guided group was 0.74 (95% CI, 0.37-1.48; *P* = .39). We observed reduced use of antipseudomonal agents (30.1%; 95% CI, 21.5%-39.9%; *P* < .001) and anti-MRSA agents (38.8%; 95% CI, 29.4%-48.9%; *P* < .001) in the Gram stain–guided group vs the guideline-based group (Table 2). Gram staining showed a sensitivity of 83.5% and specificity of 75.7% in detecting *S. aureus* (κ = 0.59) and a sensitivity of 83.0% and specificity of 60.7% in detecting gram-negative pathogens (κ = 0.43). Concordance between the results of Gram staining and culture is shown in eTable 8 in Supplement 2. There was no significant difference in coverage rates of initial antibiotic therapies between the groups (86.4% [89 of 103] vs 92.2% [95 of 103]; *P* = .18). Escalation of antibiotics according to culture results was performed in 7 patients (6.8%) in the Gram stain–guided group and 1 patient (1.0%) in the guideline-based group (*P* = .03). We observed no significant differences between the groups for all other secondary outcomes (Table 2).

### Subgroup Analyses

**Figure 2.**
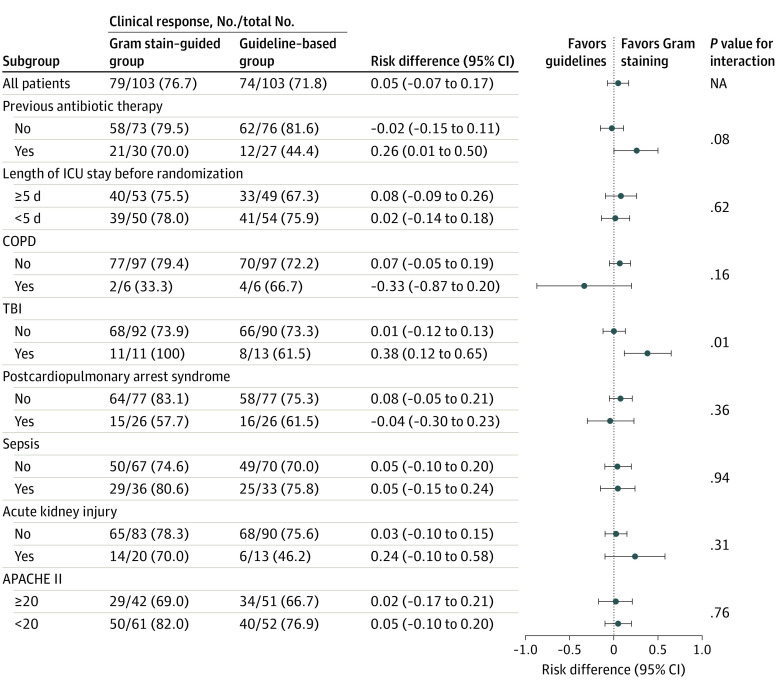
Clinical Response Rate in Prespecified Subgroups APACHE indicates Acute Physiology and Chronic Health Evaluation; COPD, chronic obstructive pulmonary disease; ICU, intensive care unit; NA, not applicable; and TBI, traumatic brain injury.

Among patients with TBI, the clinical response rate was 100% (n = 11) in the Gram stain–guided group and 61.5% (n = 13) in the guideline-based group. There was substantial heterogeneity in the effect of Gram stain–guided therapy on the clinical response in patients with TBI (risk difference, 0.38; 95% CI, 0.12-0.65; *P* = .01 for interaction). However, none of the prespecified subgroups showed substantial heterogeneity other than patients with TBI (Figure 2). Among patients with previous antibiotic therapy, the clinical response rate tended to be more favorable with the Gram stain–guided treatment than the guideline-based treatment (risk difference, 0.26; 95% CI, 0.01-0.50; *P* = .08 for interaction), although the interaction between an intervention effect and the existence of previous antibiotic therapy did not reach statistical significance. According to the length of ICU stay before randomization, the occurrence of a clinical response was similar between the 2 groups among both the patients with an ICU stay of 5 or more days and those with an ICU stay of fewer than 5 days.

### Adverse Events

There were 69 adverse events in the Gram stain–guided group and 79 in the guideline-based group (Table 3). The most common adverse event was diarrhea (27 of 103 [26.2%] vs 38 of 103 [36.9%]), followed by kidney impairment (17 [16.5%] vs 19 [18.4%]) and thrombocytopenia (16 [15.6%] vs 11 [10.7%]). Among patients with diarrhea, *C. difficile* infection was noted in 4 patients (1 [1.0%] vs 3 [2.9%]).

**Table 3.** Adverse Events

Adverse event	No. (%)	
	Gram stain–guided group (n = 103)	Guideline-based group (n = 103)
Kidney impairment	17 (16.5)	19 (18.4)
Thrombocytopenia		
Moderate	12 (11.7)	4 (3.9)
Severe	4 (3.9)	7 (6.8)
Diarrhea	27 (26.2)	38 (36.9)
*Clostridioides difficile* infection	1 (1.0)	3 (2.9)
Skin rash	3 (2.9)	3 (2.9)
Seizure	5 (4.9)	5 (4.9)

## Discussion

The GRACE-VAP trial addressed the effectiveness of Gram staining to safely restrict overuse of broad-spectrum antibiotic agents in critically ill patients with VAP. Gram stain–guided restrictive antibiotic therapy was noninferior to guideline-based broad-spectrum antibiotic therapy in patients with VAP in terms of the clinical response rate. Gram staining of endotracheal aspirates optimized the use of broad-spectrum antibiotic agents for VAP without detrimental effects on patient outcomes.

Antimicrobial resistance resulting from the overuse of antibiotics is arguably one of the greatest threats to global human health. Various procedures have been proposed to improve antimicrobial stewardship in patients with suspected pneumonia, including VAP.^24,25,26,27,28^ A major approach to optimizing antibiotic choice and minimizing unnecessary administration of antibiotics is molecular point-of-care testing. Several randomized clinical trials have been conducted to evaluate the efficacy of such tests, but the results have been conflicting.^26,27,28^ Moreover, these tests are expensive and require precision measurement instruments, and not all medical facilities can install such expensive equipment. In the GRACE-VAP trial, we used the time-honored Gram stain technique as part of the daily management of infectious diseases. We believe that the trial results are acceptable and have the potential to change the strategy of antibiotic choice worldwide.

Antibiotic choice in the guideline-based group was based on the 2016 VAP guidelines. Alternatively, the European guidelines recommend a different algorithm based on MDR risk factors for selecting antibiotic agents.^29^ Although this algorithm can certainly limit broad-spectrum antibiotic use, it might lead to an increase in inappropriate antibiotic choice.^30^ We estimated antibiotic choices and inappropriate coverage rate using the European guidelines for the patients in the guideline-based group. The European guidelines limited broad antibiotic use; however, the appropriate coverage rate also decreased considerably. If we applied the European guidelines to the patients in the guideline-based group, the lower coverage rate might result in worse outcomes for this group. Therefore, the findings should be considered robust when the European guidelines are used, but this estimation should be verified in clinical settings.

Previous antibiotic therapy can influence the results of Gram staining. Antibiotic exposure can change bacterial flora in respiratory samples, making it difficult for physicians to identify causative pathogens. An observational study found that culture positivity was reduced by 20% in blood culture specimens that were obtained during antibiotic therapy.^31^ However, in the subgroup analysis of the present trial, we did not observe any adverse effects on the clinical response attributable to previous antibiotic therapy.

### Limitations

This trial has several limitations. First, the open-label design may have influenced the behavior of the site investigator in charge. However, the schedule of clinical assessments with objective measurements was prefixed, and outcomes were assessed by independent members of the event adjudication committee, who were blinded to the group randomization. Moreover, there was no difference in the secondary end point of 28-day mortality, which was least affected by the open-label design. Second, we set the noninferiority margin at 20%, which was consistent with that in a previous trial,^32^ but the magnitude of 20% may have been too large for clinicians and the sample size was not large enough to be conclusive. The trial was conducted as planned, and the lower confidence limit of the risk difference of clinical response (−0.07) was sufficiently far from the noninferiority margin of −0.2. Moreover, the incidence of the primary outcome was observed as assumed, and all secondary outcomes and subgroup analyses supported the evidence, suggesting that the findings in the trial could be considered robust. Third, the generalizability of the trial should be considered because of the setting. Namely, the frequency of MDR GNR was low (<10%) at all participating institutions, and nonfermenters, including *Pseudomonas aeruginosa* and *Acinetobacter baumannii*, that were isolated from respiratory samples were lower than expected. Moreover, few of the trial participants had septic shock or an extremely low partial pressure of oxygen to fraction of inspired oxygen ratio. Therefore, Gram staining may not be suitable in clinical practice in patients with the most severe VAP or in hospitals or regions in which the frequency of MDR GNR is very high.

## Conclusions

This GRACE-VAP trial found that Gram stain–guided treatment compared with guideline-based treatment significantly restricted the use of broad-spectrum antibiotics without detrimental effects and yielded noninferior clinical responses in patients with VAP in the ICU. This finding reinforces the potential use of Gram staining in the critical care setting to ameliorate the spread of MDR pathogens.

## Notes

Supplement 1.
Trial Protocol

Supplement 2. **eTable 1.** Participating Institutions and Investigators **eTable 2.** Members of the Data and Safety Monitoring Board **eTable 3.** Antibiogram of Participating Institutions **eTable 4.** Microorganisms Isolated in Respiratory Samples **eTable 5.** Summary of the Results of Gram Staining **eTable 6.** Summary of Selected Antibiotic Agents **eTable 7.** Clinical Response of Participating Institutions **eTable 8.** Concordance Between Results of Gram Staining and Culture **eFigure 1.** Interventions **eFigure 2.** Twenty-Eight-Day Mortality in the Gram Stain–guided Group and Guideline-based Group

Supplement 3.
Data Sharing Statement
