# Errors in Tables 1 and 2

**DOI:** 10.1001/jamanetworkopen.2022.40335

**Published:** 2022-10-18

In the Original Investigation, “Effect of Gram Stain–Guided Initial Antibiotic Therapy on Clinical Response in Patients With Ventilator-Associated Pneumonia: The GRACE-VAP Randomized Clinical Trial,” published April 8, 2022, there were errors in the data presented in Table 1 for the Sequential Organ Failure Assessment score of the guideline-based group as well as the data for the ventilator-free days and intensive care unit–free days presented in Table 2. This article has been corrected.^1^
