# Supplementary material for: Effect of Gram Stain–Guided Initial Antibiotic Therapy on Clinical Response in Patients With Ventilator-Associated Pneumonia: The GRACE-VAP Randomized Clinical Trial
Source: JAMA Netw Open. 2022 Apr 8;5(4):e226136. doi: 10.1001/jamanetworkopen.2022.6136 (PMC8994124; doi:10.1001/jamanetworkopen.2022.6136)
Supplement: Supplement 1. — Trial Protocol [file jamanetwopen-e226136-s001.pdf]

GRam stain-guided Antibiotics ChoicE for Ventilator-Associated Pneumonia (GRACE-VAP) trial: Study Protocol for a randomized controlled trial

Principal Investigator:
Junpei Yoshimura
Division of Trauma and Surgical Critical Care
Osaka General Medical Center

Dates of Creation
July 21, 2017 Protocol First edition
March 19, 2018 Protocol Edition 1.1
April 4, 2018 Protocol Edition 1.2
June 7, 2018 Protocol Edition 1.3
July 17, 2018 Protocol Edition 1.4
April 16, 2019 Protocol Edition 1.5
May 13, 2019 Protocol Edition 1.6
January 17, 2020 Protocol Edition 1.7

Contents

1. Background 2
2. Objective 2
3. Subjects and Inclusion Criteria 2
4. Methods 3
5. Observations and Tests 5
6. Anticipated Benefits and Risks (adverse effects) 8
7. Outcome Measures (endpoints) 8
8. Discontinuation Criteria for Individual Subjects 9
9. Handling Adverse Events 9
10. Changes to the Study Protocol 9
11. Changes to and Cessation, Discontinuation, and Conclusion of the Study 9
12. Study Period 10
13. Target Number of Subjects and Rationale, Statistical Analysis Methods 10
14. Consideration of Human Rights and Protection of Personal Data 11
15. Method of Obtaining Consent 11
16. Handling of and Compensation for Health Hazards 12
17. Cost Burden Borne by Subjects 12
18. Storage of Records and Publication of Study Results 12
19. Funding and Conflict of Interest (COI) 13
20. Method of Releasing Information related to the Study 13
21. Monitoring and Supervision 13
22. Study Implementation System 13
23. Participating Facilities 13
24. Reference Materials and List of Sources 14

1. Background

The American Thoracic Society/Infectious Diseases Society of America (ATS/IDSA) Guidelines[1] recommend that physicians must first assume that the causative bacteria in cases of ventilator-associated pneumonia (VAP) is both *Staphylococcus aureus* and *Pseudomonas aeruginosa*. In particular, at facilities where the percentage of methicillin-resistant *S. aureus* (MRSA) accounts for 10% to 20% or more, administration of anti-MRSA agents are recommended. In Japan, the percentage of MRSAs has been reported to be approximately 50%.[2] Thus, to consider all inflammatory bacteria listed in the guidelines, administration of multiple broad-spectrum antibiotics would be required. However, use of these broad-spectrum antibiotics has been linked to the risk of the emergence of multi-drug resistant bacteria.[3]

Gram staining is commonly used as a method of classifying the pathogenic microorganism in cases of infectious diseases. However, in terms of VAP treatment, the clinical significance of selecting a therapeutic drug against inflammatory bacteria, which are assumed based on the results of Gram staining, is yet to be determined.[4]

After assessing the risk of resistant bacteria in VAP cases, we used Gram staining to classify inflammatory bacteria as “Gram-positive cocci (GPC) chain,” “GPC cluster,” “Gram-positive bacilli (GPB),” and “Gram-negative rods (GNR).” We then determined the therapeutic agent based on a fixed algorithm. Retrospective study of the method through comparison to a treatment algorithm created based on the ATS/IDSA Guidelines has been shown to allow less frequent use of broad-spectrum antibiotics without having a negative effect on the coverage of inflammatory bacteria.[5]

In this study we will compare antibiotic treatments recommended by the ATS/IDSA Guidelines using the algorithm to prospectively investigate their effectiveness.

2. Objective

The objective of this study is to investigate whether the therapeutic outcomes of antibiotic therapy based on Gram staining findings are non-inferior to the therapies recommended by the ATS/IDSA Guidelines by comparing the therapeutic outcomes of this method with those obtained through the use of the recommended treatments.

3. Subjects and Inclusion Criteria

(1) Subjects (2) who satisfy all inclusion criteria and (3) do not qualify for any of the exclusion criteria will be selected for this study.

(1) Subjects

The subjects will be patients undergoing respirator management
while hospitalized in the intensive care units (ICUs) of participating facilities.

(2) Inclusion criteria

① Patients aged 15 years and older at the time consent was obtained.

② Patients for whom at least 48 hours have elapsed since starting on ventilator management.

③ Patients with a modified clinical pulmonary infection score (mCPIS) of 5 or higher during ventilatory management and a clinical diagnosis of VAP (not only atelectasis or cardiac failure; other infectious agents can be ruled out).

④ Patients from whom written consent was obtained directly or from a proxy after being provided with a full description of the study and indicating that they understand that description (however, in case a proxy is unavailable prior to the study, ex post facto consent will be obtained).

mCPIS Table

	0 points	1 point	2 points
Body temp. (°C)	36.1-38.4	38.5-38.9	≤ 36 or ≥ 39
WBC (× 10^3/μL)	4-11	< 4 or > 11	< 4 or > 11 and stab cells> 500
P/F	>240 or ARDS		≤240
Xp	No infiltration	Diffuse	Localized
Sputum	None	Non-purulent	Purulent

(3) Exclusion Criteria

① Patients from whom consent could neither be obtained from the patient him/herself or from a proxy.
② Patients who are strongly opposed to therapeutic intervention.
③ Patients who are allergic to the test drug.
④ Patients who are pregnant.
⑤ Patients who have already been discharged from the ICU.
⑥ Patients whose low oxygen levels have been attributed to atelectasis or cardiac failure.
⑦ Patients who undergo antibiotic treatment at least 24 hours immediately prior to the start of randomization.
⑧ Patients the study physician has determined that entry into this study would be inappropriate.

(4) Subjects who require consent to be provided by a proxy and reasons for this

The subjects of this study are to be ICU patients, and therefore, many of them may not be able to communicate of their own free will as a result of general anesthetic treatment or a disorder of consciousness. Based on the characteristics of the illnesses that are the object of this study, we have determined that it would be difficult to conduct the study at all if we did not utilize subjects who are in this state.

In addition, as a general rule, proxies will be persons judged to be able to speak for the patient regarding the patient's wishes and benefit. When selecting proxies, consideration will be paid to the patient's family composition. The following persons will be considered:

The patient's spouse, adult children, parents, adult siblings, grandchildren, grandparents, family members, or other close relatives who reside with the patient.

4. Methods

(1) Type and Design of the Study

Multicenter, open-label, randomized, non-inferiority trial.

(2) Study Outline

The subject's or the proxy's desire to participate in this study will be confirmed and their consent obtained upon admission to the ICUs.

Patients in whom VAP with a mCIPS of 5 or higher occur and artificial ventilator management is required within 48 hours will be identified. Stochastic minimization randomization will be performed on these subjects based on the following stratification factors: facility, whether the patient suffers from chronic occlusive pulmonary disease, whether there is a history of antibiotic treatment up to the point of VAP onset, head trauma, and post-cardiac arrest syndrome. They will then be allocated to either the Gram stain-guided therapy group or the Guidelines-based therapy group. Neither group will be blinded.

Those in the Gram stain-guided therapy group will be categorized as GPC chain, GPC cluster, GPB, or GNR based on the Gram staining findings. Using sputum obtained via intratracheal aspiration prior to the start of treatment, Gram staining will be performed, and the findings evaluated by the attending physician or microbiology staff. In cases of GPC chain or GPB, a non-pseudomonal beta-lactam antibiotics will be administered in appropriate doses for at least 7 days. In cases of GPC cluster, an anti-MRSA agent will be administered in appropriate doses for at least 7 days. In cases of GNR an anti-pseudomonal agent will be administered in appropriate doses for at least 7 days. In cases in which both GPC cluster and GNR are detected, an anti-MRSA agent and an anti-pseudomonal agent will be administered in combination. Cases of other combinations will be treated using the broadest spectrum monotherapy indicated by the fixed algorithm (GPC chain + GNR: anti-pseudomonal agent, GPB + GNR: anti-pseudomonal agent, GPC chain + GPC cluster: anti-MRSA agent, GPB + GPC cluster: anti-MRSA agent).

In cases in which the microbe cannot be identified using Gram staining, combined therapy

comprising anti-MRSA agent and anti-psedomonal agent will be administered.

Subjects in the Guidelines-based therapy group will be administered combination therapy comprising anti-MRSA agent and anti-psedomonal agent for at least 7 days.

In cases in which an ant-psedomonal agent is administered, the antibiograms of each participating facility will be assessed every six months and the top 1 or 2 anti-microbials that show the highest degree of sensitivity to *P. aeruginosa* will be selected.

The anti-microbial doses will be adjusted according to renal function. Vancomycin will be subjected to therapeutic drug monitoring and adjusted so that the trough value is between 10 and 20 µg/mL.

Both groups will undergo sputum culture prior to the start of treatment. In cases in which sensitivity to the inflammatory bacteria is identified by the sputum culture, the attending physician may decide to switch the anti-microbial agent. In such cases, anti-microbial therapy will be monitored to ensure that the total period during which anti-microbial agents are administered is at least 7 days.

After the 7-day treatment period has elapsed, antibiotic treatment may be discontinued at the discretion of the attending physician.

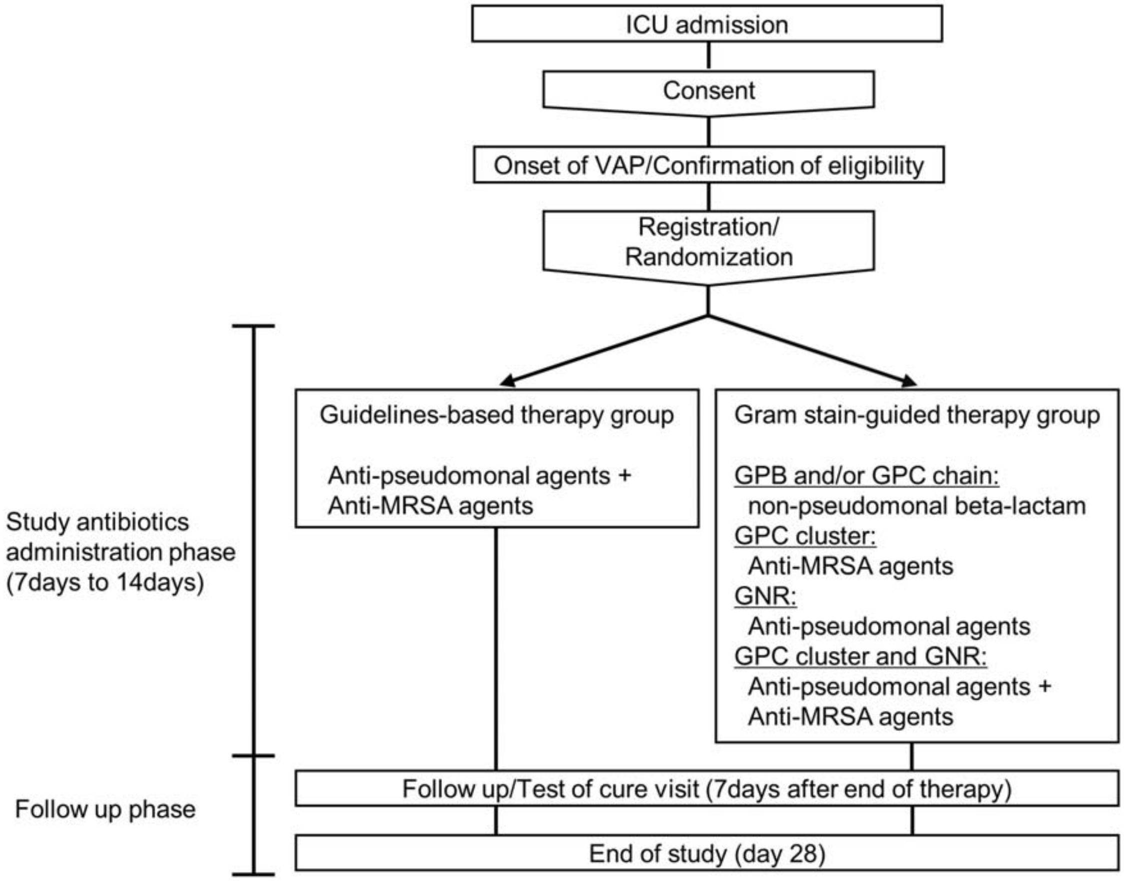

(3) Interventional study method

See the description indicated above.

(4) Restrictions regarding combination therapy

None

(5) Method of enrollment and allocation

Patients who have experienced VAP onset will be randomized using stochastic minimization procedure and will be allotted to one of the groups after their consent is obtained.

Stratification factors: Facility, whether the patient suffers from chronic occlusive pulmonary disease, whether there is a history of antibiotic treatment up to the point of VAP onset, head trauma, and post-cardiac arrest syndrome.

(6) Scheduled period during which subjects will participate in the study

All subjects will participate in an observation period lasting up to 28 days following VAP onset.

(7) Post-study support

After the study has concluded, the Principal Investigator will provide the subjects with the most suitable medical care, including the results obtained via this study.

(8) Use of specimens and data from other facilities

Yes

5. Observations and Tests

The observations and tests shown below will be implemented and the data thus obtained will be utilized in this study. All the observations and tests shown below are practiced in the course of normal medical examinations and they will be implemented at the same frequencies as normal medical examinations.

① Patient background data: Age, sex, name of illness at the time of emergency room admission, medical history (cardiac failure, chronic occlusive pulmonary disease, chronic kidney disease, diabetes, immunosuppressant use, steroid use, and antimicrobial use prior to the start of therapy), APACHE II score, SOFA score, and septic shock

② mCPIS assessment (body temperature, WBC, chest radiograph, P/F ratio, and sputum evaluation)

③ Assessment of Gram staining of sputum (Geckler's classification, type of bacterium detected)

④ Sputum culture

⑤ Blood culture

⑥ Blood test for the purpose of inflammatory response assessment (WBC, CRP, procalcitonin [PCT]*)
*PCT is listed only in those cases in which data can be obtained

⑦ Blood test for the purpose of assessing adverse events (platelet count, creatinine, AST, ALT)

⑧ Assessment of SOFA score

⑨ Duration of continuous renal replacement therapy

⑩ Assessment of clinical response

⑪ Assessment of type of antimicrobial utilized and administration period

⑫ Assessment of ventilator management period

⑬ Assessment of survival

⑭ Assessment of adverse events

TABLE: Schedule

	Admission to ICUs	Ventilator management at least 48 h.	Start	Therapy On-going					Therapy Conclusion	Day 7 after therapy conclusion	Day 28 after therapy start
			Day 1	Day 2	Day 4	Day 6	Day 8	Day 14	Day 7-14	Day 14-21	Day 28
Consent obtained	○										
Patient enrollment		○									
Patient background data obtained			○								
mCPIS assessment			○								
Gram staining findings assessment			○								
Sputum culture			○								
Blood culture			○								
Blood test for inflammatory response assessment			○	○	○	○	○	○	○	○	
Blood test for adverse event assessment			○	○	○	○	○	○	○	○	
SOFA score			○	○	○	○	○	○			
Assessment of continuous renal replacement period									○		
Assessment of clinical response										○	
Assessment of inflammatory bacteria coverage									○		
Assessment of the type, dose, period of administration of antimicrobial									○		
Assessment of ventilator management period											○

Assessment of survival											○
Assessment of adverse events				○	○	○	○	○	○	○	

6. Anticipated Benefits and Risks (adverse effects)

(1) Anticipated benefits

The trial therapy to be utilized in this study may allow selection of narrow-spectrum antibiotics, which in turn may suppress the development of multi-drug resistant bacteria. In addition, we believe the results of this study may contribute to future medical advances.

(2) Anticipated risks (adverse effects)

Anticipated serious adverse effects include anaphylactic shock, liver dysfunction, kidney dysfunction, and colitis.

In addition, the trial drugs administered to subjects in this study may include narrow-spectrum antibiotics that are ineffective against the inflammatory bacteria. However, the therapies utilized do not deviate outside the selection criteria of the drugs recommended by the ATS/IDSA Guidelines.

7. Outcome Measures (endpoints)

（1） Primary Outcome Measures

The following clinical responses will be assessed by a blinded rater:

① Antimicrobial administration is concluded by day 15 after the start of antimicrobial administration.

② Chest radiograph obtained within 24 hours following the conclusion of antimicrobial administration shows no worsening as compared to the radiograph obtained at the start of the therapy.

③ Blood test and chest radiograph are assessed on day 7 after the conclusion of treatment and show no signs or symptoms of pneumonia.

④ Antimicrobial agent administration due to pneumonia is not resumed after the treatment period.

VAP cure is determined in cases in which all the above endpoints have been satisfied and in which clinical response has been detected.

（2） Secondary Outcome Measures

・Whether anti-MRSA agent was used; whether anti-*P. aeruginosa* type antimicrobial agent was used.

・Coverage of initial antibiotic therapy: The true inflammatory bacterium is identified as the bacterium found to be in detectable amounts via pre-treatment sputum culture + amounts exceeding that amount. Cases in which the antimicrobial selected is found to be sensitive to all true inflammatory bacteria detected via sputum culture will be considered as cases in which the coverage of initial antibiotic therapy is complete.

・Whether death occurred by day 28.

・Whether the Gram staining performed by the physician matched the anticipated inflammatory bacterium identified via the Gram staining performed by the microbiology laboratory staff.

・Number of Ventilator-free days by day 28.

・Whether continuous renal replacement therapy was performed.

・Period of antimicrobial therapy.

・Whether de-escalation occurred.

・Whether the blood culture results match the sputum culture results.

・Renal dysfunction onset: During the period up to day 7 after the conclusion of treatment, creatinine increase of at least 1.5× over baseline will be defined as renal dysfunction

・Thrombocytopenia: Moderate cases defined as platelet count decrease between at least 30% and less than 50% of baseline up to 7 days after antibiotic administration conclusion and severe cases defined as decreases of 50% or more.

・Onset of other adverse events: Illnesses such as liver damage, diarrhea, skin rash, and convulsions whose symptoms occur by day 7 after the conclusion of treatment.

8. Discontinuation Criteria for Individual Subjects

(1) Support at discontinuation of the study

The Principal Investigator or Co-Investigators (hereinafter "Investigators") will discontinue the participation of a subject in this study when it is determined that it is impossible for the subject in question to continue participating in the study for one or more of the reasons listed below. In such a case, the subject will be informed of the reason for discontinuation of participation when such a description is considered necessary. Furthermore, after discontinuation, the subject will be provided with treatment that will remove any further risk to the subject.

(2) Discontinuation criteria

① The subject requests that his or her participation in the study be discontinued or the subject rescinds consent to participate in the study.

② The entire study itself is discontinued.

9. Handling Adverse Events

(1) Support provided to subjects when adverse events occur

When adverse events occur, the Investigators will immediately provide the subject in question with appropriate treatment and record the details in the subject's medical record and case report. In cases in which interventional procedures are discontinued or cases in which treatment of the adverse event is required, the subject will be informed of these facts.

(2) Reporting serious adverse events

Serious adverse events are defined as below in accordance with Article 273 of the Pharmaceutical Affairs Act Enforcement Regulations:

1) Events that may cause death.
2) Events that require hospitalization or extension of hospital stay for the purpose of treatment.
3) Events that cause permanent or serious disability/dysfunction.
4) Events that cause congenital diseases/abnormalities or diseases/abnormalities in descendants.

The Principal Investigator will report all serious events that occur during the study period and serious events detected after the conclusion (discontinuation) of the study that are suspected of being related to the interventional procedures performed during the study to the President via the Independent Clinical Research Bureau. Reports in such cases will comprise an Initial Report ("emergency report") and a Secondary Report ("detailed report"), in accordance with the Procedures of the Independent Clinical Research Bureau.

(3) Reporting significant adverse events

N/A

(4) Other adverse events

Other adverse events will be handled as follows: The investigators will record the event on the subject's medical record and case report as appropriate.

10. Changes to the Study Protocol

The prior approval of the Institutional Review Board for the Protection of Human Subjects in Research (hereinafter referred to as the "Institutional Review Board") is required in case changes or revisions are made to the study protocol or the study description and consent form.

11. Changes to and Cessation, Discontinuation, and Conclusion of the Study

(1) Changes to the study

The prior approval of the Institutional Review Board is required in case changes are made to the study protocol or the study description and consent form.

(2) Cessation and discontinuation of the study

The Investigators will consider whether to continue the study in the following cases:

① When important information regarding the quality, safety, or effectiveness of materials and instruments (e.g. the test drug) used in invasive or interventional procedures is obtained.

② When it is determined that it would be extremely difficult to reach the target number of subjects because of difficulties in including subjects.

③ When the objective of the study is achieved prior to reaching the target number of subjects or prior to the end of the study period.

④ When the Institutional Review Board directs that changes are to be made to the protocol or other elements of the study but it is determined that it would be difficult to carry out such directions.

In case the Institutional Review Board recommends or directs that the study be discontinued, the Principal Investigator will discontinue the study. Further, when cessation or discontinuation of the study has been decided upon, the President will immediately be informed in writing of the reason or reasons for this decision.

(3) Conclusion of the study

Once the study has been concluded, the Principal Investigator will promptly inform the President that the study has been concluded.

12. Study Period

The study period is scheduled to begin in April 2018 and continue for three years. Once the target number of subjects has been reached, the study will conclude.

13. Target Number of Subjects and Rationale, Statistical Analysis Methods

(1) Target number of subjects and the rationale for setting that target number

When the non-inferiority margin is 20%, the number of target subjects is 200 (Standard group: 100, Gram-stain group: 100).

Rationale

Based on the results of observational studies, the clinical response cure rate in the Standard group is estimated to be 67.8%. Further, we have taken into consideration the non-inferiority margins utilized by past non-inferiority trials in the field of intensive care.[6] The Type I error is set at 5% and Type II error is set at 20%. The non-inferiority margin is set at 20%. Thus, the number of subjects required for a detection rate of 80% is 172. As we have estimated that approximately 10% of the subjects will be disqualified owing to missing data and other problems, we have set the target number of subjects as 200.

(2) Statistical analysis methods

Patient background data will be obtained via descriptive responses. The Gram stain-guided therapy group and the Guidelines-based therapy group will be compared using the following items: clinical response, anti-MRSA agent usage rate, anti-*P. aeruginosa* activity agent usage rate, inflammatory bacteria coverage rate, incidence of renal dysfunction, incidence of thrombocytopenia, incidence of other adverse events, and mortality rate by day 28.

Further, the same inter-group comparisons will be made for the following sub-groups: Sex, head trauma, post-resuscitation encephalopathy, chronic occlusive pulmonary disease, and history of antibiotic use during hospitalization.

The sub-analyses are at the present time scheduled to include the following items. However, the details will be examined in the future by the Institutional Review Boards of the participating institutions.

・Assessment of resistant bacteria risk as a result of patient background characteristics.

・Inter-facility comparison of inflammatory bacteria coverage rates.

・Comparisons of clinical responses, mortality rate by day 28, whether ventilator-free days by de-escalation occurred

・Comparison of the matching rate between the physician-performed and microbiology laboratory staff-performed Gram staining for anticipated inflammatory bacteria.

・The following comparisons will be made for immunosuppressed patients (Gram-stain group and Standard group): clinical response, mortality rate by day 28, and number of ventilator-free days.

・The following comparisons will be made between patients who used vancomycin and patients who used linezolid: clinical response, mortality rate by day 28, number of ventilator-free days, and adverse event incidence.

・Comparisons of continuous renal replacement therapy or no therapy and period of antibiotic therapy.

・Comparisons of Gram-stained flat epithelial cells (yes vs. no) and clinical response, mortality rate at day 28, and number of ventilator-free days.

・Match rate between blood culture results and sputum culture results.

14. Consideration of Human Rights and Protection of Personal Data

All Investigators associated with this study will abide by the Declaration of Helsinki (October 2013 revision) and the Ethical Guidelines for Medical and Health Research Involving Human Subjects (partial revision on February 28, 2017; hereinafter referred to as the "Ethical Guidelines").

When handling samples related to this study, a number that is unrelated to the subject's personal data will be assigned to the sample for the purpose of sample management and full consideration will be given to the protection of the subject's privacy. When such samples and other materials are sent to the Research Bureau and other related facilities this number will be utilized to ensure that full consideration is given to the prevention of the subject's personal data leaking outside the hospital where the subject is admitted as a patient. When publicizing the results of this study, no data that could be used to identify individual subjects will be included. The samples and other materials collected from the subjects as part of this study will not be used for any purpose other than this study.

15. Method of Obtaining Consent

The Investigators will provide the subjects (or their proxies in cases in which a proxy was required) with a copy of the Study Description Form used to obtain the approval of the Institutional Review Board. They will be provided with full written and oral descriptions of the study, and their consent to participate in this study of their own free will be obtained in writing.

Considering the medical care provided in the ICU and the emergency nature of VAP onset, patients admitted to the ICU will be counselled regarding the possibility of tracheal intubation and the associated risk of VAP, and their prior consent to participate in this study will be obtained as soon as possible after admission to the ICU. In cases in which the patient satisfies the inclusion criteria and is therefore enrolled in this study, their intention to participate in this study will be re-confirmed after treatment. If, after admission to the ICU, a patient is found not to satisfy the inclusion criteria, the consent form they provided, and all related medical data will be discarded upon the patient's discharge from the ICU.

When information is obtained that may have an effect on the subjects' consent or changes are made to the study protocol that may have an effect on the subjects' consent, this information will be provided promptly to the subjects by the Investigators. The Investigators will then confirm the subjects' intent to participate in this study and will revise the Description and Consent Form accordingly after obtaining the prior approval of the Institutional Review Board, and they will then obtain additional consent from the subjects.

The Description and Consent Form will include the following:

① The fact that the name of the study and the study itself were approved by the Head of the participating facility.

② The name of the participating facility and the name of the Principal Investigator (including the names of joint-research facilities and the Principal Investigator)

③ The objectives and significance of the study.

④ The study methods (including the purpose of using the materials and data collected from the subjects) and the study period.
⑤ The reason the subject was selected.
⑥ The burden to be borne by the subject and the anticipated benefits and risks.
⑦ The fact that a subject may drop out of the study at any time regardless of having granted consent to participate or continue in the study.
⑧ The fact that subjects will not experience any disadvantage as a result of not consenting to participate or continue in the study or rescinding such consent.
⑨ The manner in which information related to the study will be publicized.
⑩ The fact that study subjects or their proxies may obtain or read the study protocol and materials related to the study methods and how they may obtain and read these materials.
⑪ Handling of personal information and data (when anonymized data is used, this includes the method of anonymization).
⑫ How materials and data are to be stored and discarded.
⑬ Funding, etc. of the study, conflicts of interest related to the participating facilities and any income received by individuals, and conflicts of interest associated with the researchers.
⑭ Consultations, etc. provided by the researchers and related persons.
⑮ Financial burdens or rewards provided to the subjects.
⑯ How the study results (including incidental findings) will be handled in case important information related to the health of the subjects and the genetic characteristics of their descendants may be obtained via this study.
⑰ Whether compensation will be provided in case a subject’s health is negatively affected by this study and the details of this compensation.
⑱ Regarding materials and data obtained from the subjects: In case these may be used for the purpose of future studies that have not yet been identified as of the time consent is obtained from the subjects or in case they may be provided to other facilities, the details will be provided at the time subjects are provided with an explanation and their consent is obtained.

16. Handling of and Compensation for Health Hazards

Measures will be taken when important information regarding the quality, safety, or effectiveness of materials and instruments (e.g., the test drug) used in invasive or interventional procedures is obtained. As the medical care provided in conjunction with this study is covered by the NHI system of Japan, when treatment or tests are required, the normal procedures stipulated by the subject’s health insurance or the physician’s liability insurance will be performed. An explanation of the above details will be provided to the subjects to ensure that these procedures are understood.

17. Cost Burden Borne by Subjects

As the procedures performed in conjunction with this study are covered by the NHI system of Japan, no cost burden associated with this study will be borne by the study subjects.

18. Storage of Records and Publication of Study Results

1.) Materials and data obtained in conjunction with this study

Materials and data obtained in conjunction with this study will be stored in a high-security database for a period of five years after the conclusion or discontinuation of this study. Access will be limited to the Osaka General Medical Center and data centers that have been requested to handle the materials and data by the Osaka General Medical Center. After this five-year period has elapsed, all personal data will be carefully discarded. The materials and data will be stored after removal of all information, such as name and date of birth, that could be used to identify individual subjects.

2.) Materials related to data utilized in this study

The Principal Investigator will store important documents (copies of applications, notices from the Director, copies of all types of applications and reports, consent forms, and all other documents and records required to guarantee the reliability of the data) related to the implementation of this study, etc. for a period of five years from the conclusion or discontinuation of the study. Thereafter, these materials will be discarded with particular care taken to avoid the disclosure of personal information.

The Investigators will publicize the results of this study through presentations at academic societies and similar forums.

19. Funding and Conflict of Interest (COI)

This study will be conducted using medical fees and research fees provided by the facilities with which the Principal Investigator is affiliated. The Investigators will report to the COI Management Review Board all required items and undergo the required screening and approval process.

20. Method of Releasing Information related to the Study (registration of the study protocol and publication of the study results)

This study will be registered with the Clinical Trials.gov and the UMIN Database. Furthermore, the results obtained in conjunction with this study will be presented at international academic conferences and publicized as papers published in journals focusing on the field of intensive care. In all cases, the results that are publicized will be subject to statistical processing to ensure that no personal information associated with the study subjects is released.

21. Monitoring and Supervision

(1) Monitoring

When required, the Monitor will examine the records associated with this study (case reports, raw data, and other related materials) to confirm the integrity of the data, its accuracy and consistency, and compliance with ethical and other guidelines. The Monitor will confirm the content of the case reports and ensure the integrity of the study.

The Monitor will report to the Principal Investigator any deviations from the study protocol, all procedures, and applicable regulations. The Monitor will ensure that appropriate measures are implemented to prevent repeats of the deviations discovered and ensure that these measures are recorded.

The Principal Investigator agrees to cooperate with the Monitor to ensure that all problems discovered through the monitoring process are handled and recorded.

(2) Supervision

This study may be subject to supervision. Inspections of the sites where the study is being conducted and reviews of records related to the study will be conducted to assess the way the study is being implemented as well as compliance with the study protocol and all relevant regulations. When supervision is conducted, the Supervisor must report the results of the supervisory activities to the Principal Investigator and the Chairperson.

22. Study Implementation System

Representative Facility

Osaka General Medical Center

Organization

○ Jumpei Yoshimura Physician, Division of Trauma and Surgical Critical Care, Osaka General Medical Center
Satoshi Fujimi Manager of Division of Trauma and Surgical Critical Care
Kazuma Yamakawa Vice Manager of Division of Trauma and Surgical Critical Care
Yasutaka Nakahori Vice Manager of Division of Trauma and Surgical Critical Care

Naoki Nakamoto Chief Physician, Division of Trauma and Surgical Critical Care
Yutaka Umemura Medical Chief, Division of Trauma and Surgical Critical Care
Atsushi Watanabe Physician, Division of Trauma and Surgical Critical Care
Hiroshi Ito Physician, Division of Trauma and Surgical Critical Care
Takeshi Nishida Physician, Division of Trauma and Surgical Critical Care
Keishi Okamoto Physician, Division of Trauma and Surgical Critical Care
Naoki Okada Physician, Division of Trauma and Surgical Critical Care
Yo Hidaka Physician, Division of Trauma and Surgical Critical Care
Naohito Meguro Physician, Division of Trauma and Surgical Critical Care
Hiroki Matsuda Support Physician, Division of Trauma and Surgical Critical Care
Takeyuki Kiguchi Support Physician, Division of Trauma and Surgical Critical Care
Takahiro Kinoshita Support Physician, Division of Trauma and Surgical Critical Care
(○=Principal Investigator)

Research Bureau

Division of Trauma and Surgical Critical Care, Osaka General Medical Center
Address: 3-1-56 Bandai-higashi, Sumiyoshi, Osaka
Tel: 06-6692-1201

Data Center

Institute for Clinical Effectiveness (ICE)
Address: 403 Shimo-maruya-cho, Oike-sagaru, Kawahara-machidori, Nakagyo-ku, Kyoto
Tel: 075-778-5054
Contact person: Makiko Ootori

Statistical Analysis Supervisor

Takeshi Morimoto, Department of Clinical Epidemiology, Hyogo College of Medicine

23. Participating Facilities

Acute & Critical Care Center, Nagasaki University Hospital, Shuhei Yamano
Department of Emergency and Critical Care Medicine, Kansai Medical University Hospital, Hiroki Takahashi
Department of Emergency and Critical Care Medicine, Kansai Medical University Medical Center, Masahiro Kawada
Department of Emergency and Critical Care Medicine, Saga University Hospital, Hiroyuki Koami
Department of Emergency and Critical Care Medicine, Hitachi General Hospital, Kensuke Nakamura
Department of Trauma, Critical Care Medicine and Burn Center, Japan Community Healthcare Organization Chukyo Hospital, Akinori Osuka
Department of Emergency Medicine, Ebina General Hospital, Takeshi Yamagiwa
Department of Emergency and Critical Care Medicine, Wakayama Medical University Hospital, Kyohei Miyamoto
Department of Emergency Medicine, University of Ryukyus Hospital, Takayuki Hirara
Department of Emergency and Critical Care Medicine, Sapporo City General Hospital, Akira Kodate
Tajima Emergency and Critical Care Medical Center
Makoto Kobayashi

24. Reference Materials and List of Sources

1. Andre C. Kalil, Mark L. Metersky, Michael Klompas, *et al.*: Management of adults with hospital-acquired and ventilator-associated pneumonia: 2016 clinical practice guidelines by the Infectious Diseases Society of America and the American Thoracic Society. **Clin Infect Dis. 63:** e61-e111. 2016
2. Ministry of Health, Labour and Welfare: Nosocomial infection countermeasure surveillance,

Public data on all in-hospital patients (2014 report). 2015
3. Goossens H, Ferech M, Vander Stichele R, *et al.*: Outpatient antibiotic use in Europe and association with resistance: a cross-national database study. **Lancet. 365:** 579-587. 2005
4. Gottesman T, Yossepow O, Lerner E, *et al.*: The accuracy of Gram stain of respiratory specimens in excluding *Staphylococcus aureus* in ventilator-associated pneumonia. **J Crit Care. 29:** 739-742. 2014
5. Yoshimura J, Kinoshita T, Yamakawa K, *et al.*: Impact of Gram stain results on initial treatment selection in patients with ventilator-associated pneumonia: a retrospective analysis of two treatment algorithms: **Crit Care. 21:**156. 2017.
6. Vance G. Fowler Jr., Helen W. Boucher, G. Ralph Corey, *et al.*: Daptomycin versus standard therapy for bacteremia and endocarditis caused by *Staphylococcus aureus*: **N Engl J Med. 355:** 653-665. 2006.

History of Changes

Date of Change	Responsible Party	Details of change
2017/7/21<br>Ver.1.0	Jumpei Yoshimura	First edition confirmed
2018/3/19<br>Ver.1.1	Jumpei Yoshimura	Following site investigators at Osaka General Medical Center were added.<br>Hiroki Matsuda, Naoki Nakamoto, Atsushi Watanabe, Takeyuki Kiguchi<br>Following participating facility was excluded.<br>Kagawa University Hospital<br>Following participating facility was added<br>University of Ryukyus Hospital
2018/4/4<br>Ver.1.2	Jumpei Yoshimura	Following exclusion criteria was added.<br>Patients the study physician has determined that entry into this study would be inappropriate.<br>Following site investigators at Osaka General Medical Center were added.<br>Hiroshi Ito, Takayuki Shiwaku, Keishi Okamoto, Hisaya Domi
2018/6/7<br>Ver.1.3	Jumpei Yoshimura	Following participating facility was excluded.<br>Hokkaido University Hospital
2018/7/17<br>Ver.1.4	Jumpei Yoshimura	Following participating facility was added.<br>Tajima Emergency and Critical Care Medical Cente<br>Japanese Red Cross Society Kyoto Daini Hospital
2019/4/16<br>Ver.1.5	Jumpei Yoshimura	Following site investigators at Osaka General Medical Center were added.<br>Yasutaka Nakahori, Yutaka Umemura, Takeshi Nishida, Naoki Okada, Yo Hidaka, Naohito Meguro<br>Following site investigators at Osaka General Medical Center were excluded.<br>Takayuki Shiwaku, Hisaya Domi<br>Following participating facility was added.<br>National Defense Medical College Hospital<br>Following participating facility was excluded.<br>Japanese Red Cross Society Kyoto Daini Hospital
2019/5/13<br>Ver.1.6	Jumpei Yoshimura	Following participating facility was added.<br>Sapporo City General Hospital
2020/1/17<br>Ver. 1.7	Jumpei Yoshimura	Following participating facility was excluded.<br>National Defense Medical College Hospital<br>Final version confirmed

July 11, 2018 Analysis plan Edition 0.1
October 4, 2019 Analysis plan Edition 1.0

GRACE-VAP trial
Statistical analysis plan

Study statistician
Takeshi Morimoto
Department of Clinical Epidemiology
Hyogo College of Medicine

Ver.1.0

Statistical analysis plan

1. Definition of terms……………………………………..……………………………………..3
1.1. Clinical response and treatment failure…….………………………………………………..3
1.2. The coverage of initial antibiotic therapy…………………………………………………....3
1.3. De-escalation…………………………..……………………………………………………3
1.4. Abbreviation…………………………………………………………………………………3
2. Methods…………………………………………………………………………………………..4
2.1. Objective…………………………………………………………………………………..4
2.2. Type and design of the study………………………...…………………………………………4
2.3 Target number of subjects and rationale…………………………………………………………..8
3. Handling of cases and data…………………………………………………………..………......9
3.1. Case management…...9
3.2. Data management..9
4. Details of statistical analysis plan..10
4.1. Investigation of backgrounds..10
4.2. Primary analysis of effectiveness...11
4.3. Secondary analysis of effectiveness...11
4.4. Safety analysis...13
4.5. Subgroup analysis..14
4.6. Other...14
5. References..14
6. History of changes...14

1.Definition of terms

1. 1. Clinical Response and Treatment failure

Clinical response is determined in cases in which all the below endpoints have been satisfied. Treatment failure is determined in the other cases.

1) Antimicrobial administration is concluded by day 15 after the start of antimicrobial administration.

2) Chest radiograph obtained within 24 hours following the conclusion of antimicrobial administration shows no worsening as compared to the radiograph obtained at the start of the therapy.

3) Blood test and chest radiograph are assessed on day 7 after the conclusion of treatment and show no signs or symptoms of pneumonia.

4) Antimicrobial agent administration due to pneumonia is not resumed after the treatment period.

1. 2. The coverage of initial antibiotic therapy

The true inflammatory bacterium is identified as the bacterium found to be in detectable amounts via pre-treatment sputum culture + amounts exceeding that amount. Cases in which the antimicrobial selected is found to be sensitive to all true inflammatory bacteria detected via sputum culture will be considered as cases in which the coverage of initial antibiotic therapy is complete.

1. 3. De-escalation

The de-escalation is determined as follow:

1) replacing a broad-spectrum antibiotic with another with narrow-spectrum
2) stopping the element of antibiotic combination therapy.

1. 4. Abbreviation

VAP：Ventilator-associated pneumonia
MRSA: Methicillin-resistant Staphylococcus aureus
mCPIS: modified Clinical pulmonary infection score
APACHE II: Acute physiology and chronic health evaluation II
SOFA: Sequential organ failure assessment
CRP: C-reactive protein
PCT: Procalcitonin
AST: Aspartate aminotransferase
ALT: Alanine aminotransferase

2.Methods

2. 1. Objective

The objective of this study is to investigate whether the therapeutic outcomes of antibiotic therapy based on Gram staining findings are non-inferior to the therapies recommended by the ATS/IDSA Guidelines by comparing the therapeutic outcomes of this method with those obtained through the use of the recommended treatments.。

2. 2. Type and Design of the Study

2.2.1.Outcome Measures

2.2.1.1.Primary Outcome Measures

Clinical Response of VAP

2.2.1.2.Secondary Outcome Measures

・Whether anti-MRSA agent was used; whether anti-*P. aeruginosa* type antimicrobial agent was used.

・Coverage of initial antibiotic therapy: The true inflammatory bacterium is identified as the bacterium found to be in detectable amounts via pre-treatment sputum culture + amounts exceeding that amount. Cases in which the antimicrobial selected is found to be sensitive to all true inflammatory bacteria detected via sputum culture will be considered as cases in which the coverage of initial antibiotic therapy is complete.

・Whether death occurred by day 28.

・Whether the Gram staining performed by the physician matched the anticipated inflammatory bacterium identified via the Gram staining performed by the microbiology laboratory staff.

・Number of Ventilator-free days by day 28.

・Whether continuous renal replacement therapy was performed.

・Period of antimicrobial therapy.

・Whether de-escalation occurred.

・Whether the blood culture results match the sputum culture results.

・Renal dysfunction onset: During the period up to day 7 after the conclusion of treatment, creatinine increase of at least 1.5× over baseline will be defined as renal dysfunction

・Thrombocytopenia: Moderate cases defined as platelet count decrease between at least 30% and less than 50% of baseline up to 7 days after antibiotic administration conclusion and severe cases defined as decreases of 50% or more.

・Onset of other adverse events: Illnesses such as liver damage, diarrhea, skin rash, and convulsions whose symptoms occur by day 7 after the conclusion of treatment.

2.2.2.Study design

Multicenter, open-label, randomized, non-inferiority trial.

2.2.3.Study Outline

The subject’s or the proxy’s desire to participate in this study will be confirmed and their consent obtained upon admission to the ICUs.

Patients in whom VAP with a mCIPS of 5 or higher occur and artificial ventilator management is required within 48 hours will be identified. Stochastic minimization randomization will be performed on these subjects based on the following stratification factors: facility, whether the patient suffers from chronic occlusive pulmonary disease, whether there is a history of antibiotic treatment up to the point of VAP onset, head trauma, and post-cardiac arrest syndrome. They will then be allocated to either the Gram stain-guided therapy group or the Guidelines-based therapy group. Neither group will be blinded.

Those in the Gram stain-guided therapy group will be categorized as GPC chain, GPC cluster, GPB, or GNR based on the Gram staining findings. Using sputum obtained via intratracheal aspiration prior to the start of treatment, Gram staining will be performed, and the findings evaluated by the attending physician or microbiology staff. In cases of GPC chain or GPB, a non-pseudomonal beta-lactam antibiotics will be administered in appropriate doses for at least 7 days. In cases of GPC cluster, an anti-MRSA agent will be administered in appropriate doses for at least 7 days. In cases of GNR an anti-pseudomonal agent will be administered in appropriate doses for at least 7 days. In cases in which both GPC cluster and GNR are detected, an anti-MRSA agent and an anti-pseudomonal agent will be administered in combination. Cases of other combinations will be treated using the broadest spectrum monotherapy indicated by the fixed algorithm (GPC chain + GNR: anti-pseudomonal agent, GPB + GNR: anti-pseudomonal agent, GPC chain + GPC cluster: anti-MRSA agent, GPB + GPC cluster: anti-MRSA agent).

In cases in which the microbe cannot be identified using Gram staining, combined therapy comprising anti-MRSA agent and anti-pseudomonal agent will be administered.

Subjects in the Guidelines-based therapy group will be administered combination therapy comprising anti-MRSA agent and anti-pseudomonal agent for at least 7 days.

In cases in which an ant-pseudomonal agent is administered, the antibiograms of each participating facility will be assessed every six months and the top 1 or 2 anti-microbials that show the highest degree of sensitivity to *P. aeruginosa* will be selected.

The anti-microbial doses will be adjusted according to renal function. Vancomycin will be subjected to therapeutic drug monitoring and adjusted so that the trough value is between 10 and 20 µg/mL.

Both groups will undergo sputum culture prior to the start of treatment. In cases in which sensitivity to the inflammatory bacteria is identified by the sputum culture, the attending physician may decide to switch the anti-microbial agent. In such cases, anti-microbial therapy will be monitored to ensure that the total period during which anti-microbial agents are administered is at least 7 days.

After the 7-day treatment period has elapsed, antibiotic treatment may be discontinued at the discretion of the attending physician.

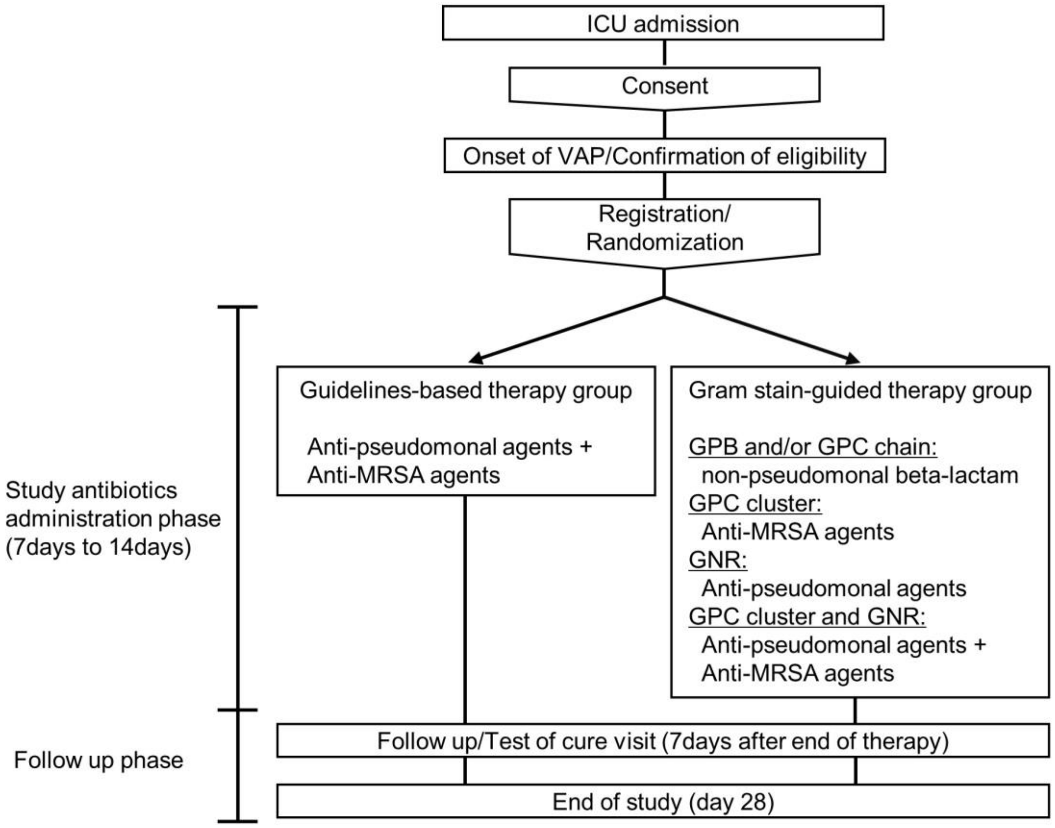
ICU admission
Consent
Onset of VAP/Confirmation of eligibility
Registration/
Randomization
Study antibiotics
administration phase
(7days to 14days)
Guidelines-based therapy group
Anti-pseudomonal agents +
Anti-MRSA agents
Gram stain-guided therapy group
GPB and/or GPC chain:
non-pseudomonal beta-lactam
GPC cluster:
Anti-MRSA agents
GNR:
Anti-pseudomonal agents
GPC cluster and GNR:
Anti-pseudomonal agents +
Anti-MRSA agents
Follow up phase
Follow up/Test of cure visit (7days after end of therapy)
End of study (day 28)

2.2.4.Schedule

	Admission to ICUs	Ventilator management at least 48 h.	Start	Therapy On-going					Therapy Conclusion	Day 7 after therapy conclusion	Day 28 after therapy start
			Day 1	Day 2	Day 4	Day 6	Day 8	Day 14	Day 7-14	Day 14-21	Day 28
Consent obtained	○										
Patient enrollment		○									
Patient background data obtained			○								
mCPIS assessment			○								
Gram staining findings assessment			○								
Sputum culture			○								
Blood culture			○								
Blood test for inflammatory response assessment			○	○	○	○	○	○	○	○	
Blood test for adverse event assessment			○	○	○	○	○	○	○	○	
SOFA score			○	○	○	○	○	○			
Assessment of continuous renal replacement period									○		
Assessment of clinical response										○	
Assessment of inflammatory bacteria coverage									○		

Assessment of the type, dose, period of administration of antimicrobial									○		
Assessment of ventilator management period											○
Assessment of survival											○
Assessment of adverse events				○	○	○	○	○	○	○	

2. 3. Target Number of Subjects and Rationale

2.3.1.Target number of subjects

The number of target subjects is 200 (Standard group: 100, Gram-stain group: 100).

2.3.2.The rationale for setting the target number

Based on the results of observational studies, the clinical response cure rate in the Standard group is estimated to be 67.8%[1]. Further, we have taken into consideration the non-inferiority margins utilized by past non-inferiority trials in the field of intensive care.[2] The Type I error is set at 5% and Type II error is set at 20%. The non-inferiority margin is set at 20%. Thus, the number of subjects required for a detection rate of 80% is 172. As we have estimated that approximately 10% of the subjects will be disqualified owing to missing data and other problems, we have set the target number of subjects as 200.

3.Handling of Cases and Data

3. 1. Case Management

3.1.1.Analysis Set

3.1.1.1.Safety Analysis Set (SAS)

SAS: Patient population that participated in the study and was assigned to one of the treatment groups (regardless of whether it was administered the test drug)

3.1.1.2.Effectiveness Analysis Set

FAS (Full Analysis Set)

Patient population that participated in this study, was assigned into one of the treatment groups and then after receiving antibiotic treatment for at least the duration of the treatment period, was able to be evaluated using the primary evaluation items specified in the study protocol (regardless of the type of antibiotic).

This corresponds to the ITT (Intention-to-treat population).

PPS (Per Protocol Set)

Patient population that participated in the study, was assigned to one of the treatment groups, received the treatment specified in the study protocol and was able to be evaluated using the primary evaluation items.

FAS and PPS were used as the analysis groups for the primary and secondary analyses, respectively.

3. 2. Data Management

3.2.1.Items Related to Time Lag

① Blood test during observation period, SOFA score

The value taken from 0 to 1 day after the evaluation date was considered the measurement value during the observation period.

② Evaluation of clinical response

This includes data taken between 3 days before and 7 days after the evaluation date. In situations where there was overlapping data, data taken on a date that was close to the date when a clinical effect was evaluated, was used.

4.Details of Statistical Analysis Plan

4. 1. Investigation of Backgrounds

4.1.1.Patient flow diagram

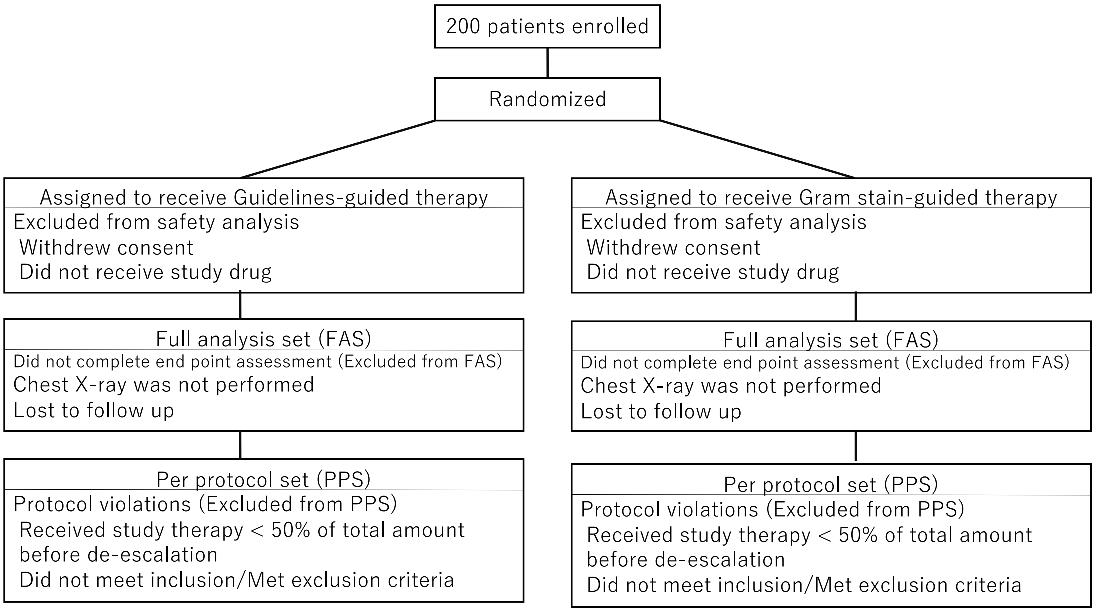

∗ A breakdown of the number of cases is shown separately (Figure 2).

4.1.2.Background Information

Items: The following patient backgrounds

Analysis Group(s): FAS

Purpose: The purpose is to determine the ranking of the sample by compiling and classifying the background of each group.

Indicated by: Categorical data: number of cases, percentages

Continuous data: averages, S.D., maximum, minimum, median

The rankings of the samples were determined by compiling and classifying the following patient backgrounds as a whole and by group:

age, sex, name of disease when admitted into the ICU (trauma, sepsis, after cardiopulmonary resuscitation, etc.), mCPIS, presence or absence of complications (chronic heart failure, maintenance dialysis, cirrhosis of the liver, chronic respiratory disease, diabetes, immunosuppressive status), history of antibiotic treatment from the period of initial hospitalization to assignment into a treatment group, ICU admission period before entry (≥5 days, <5 days), presence or absence of sepsis or septic shock and acute kidney as well as APACHE II and SOFA.

4.2. Primary Analysis of Effectiveness

4.2.1.Comparison of clinical response between groups

Item(s) : Clinical response
Analysis Group(s): FAS and PPS
Analysis : Normal theory test for binomial proportions
Time Period : 7 days after completion of pneumonia treatment
Purpose : Verification of non-inferiority and superiority
Significance Level: One-sided 2.5%
Estimations : Two-sided 95% confidence interval of differences between groups

Determination of non-inferiority and superiority:

In a normal theory test, two-sided 95% confidence intervals for the difference ($\mu 2 - \mu 1$) between the ratios of the clinical responses are calculated. If the upper limit of the confidence interval does not exceed 20%, non-inferiority is verified. Furthermore, after non-inferiority is verified, if it is confirmed that the upper limit of the confidence interval does not exceed 0%, then it is determined that superiority has been verified. In this analysis, H1: $\mu 1 > \mu 2 - 20\%$ was tested against H0: $\mu 1 \leqq \mu 2 - 20\%$. Furthermore, during the verification of superiority, H1: $\mu 1 > \mu 2$ was tested against H0: $\mu 1 \leqq \mu 2$. Here, $\mu 1$ is the ratio of the Gram-stain group and $\mu 2$ is the ratio of the guideline group.

Indicated by : Number of cases and percentages

4.3. Secondary Analysis of Effectiveness

4.3.1.Items related to antibiotic treatment

4.3.1.1.Comparison of anti-MRSA agent use between groups

Item(s) : Inter-group comparison of anti-MRSA agent use
Analysis Group(s): FAS
Test(s) : χ^2 test
Significance Level: Two-sided 5%
Estimations : Dose rates and 95% confidence intervals
Indicated by : Number of cases and percentages

4.3.1.2.Comparison of anti-pseudomonal agent use between groups

Item(s) : Inter-group comparison of anti-pseudomonal agent use
Analysis Group(s): FAS
Test(s) : χ^2 test
Significance Level: Two-sided 5%
Estimations : Dose rate and 95% confidence intervals
Indicated by : Number of cases and percentages

4.3.1.3.Comparison of coverage of initial antibiotic therapy between groups

Item(s) : Inter-group comparison of antibiotic treatment periods
Analysis Group(s): FAS
Test(s) : Wilcoxon rank-sum test
Significance Level: Two-sided 5%
Estimations : Medians and IQRs (interquartile range) of antibacterial treatment periods)
Indicated by : Median and IQR(interquartile range) values

4.3.1.4.Comparison of antibiotic treatment periods between groups

Item(s) : Inter-group comparison of antibiotic treatment periods
Analysis Group(s): FAS
Test(s) : Wilcoxon rank-sum test
Significance Level: Two-sided 5%
Estimations : Medians and IQRs (interquartile range) of antibacterial treatment periods)
Indicated by : Median and IQR(interquartile range) values

4.3.1.5.Comparison of de-escalation between groups

Item(s) : Inter-group comparison of de-escalation
Analysis Group(s): FAS
Test(s) : χ^2 test
Significance Level: Two-sided 5%
Estimations : De-escalation rates and 95% confidence intervals
Indicated by : Number of cases and percentages

4.3.2.Items Related to Medical Test Values

4.3.2.1. Items related to blood test values

Item(s) : Inflammation markers (CRP, PCT)
Analysis Group(s) : FAS and PPS
Scale : Measured value
Time Period : Observation Period
Test(s) : (Inter-group) Two-sample t-test of differences for the amount of change or rate of change during the observation period
(Intra-group) One-sample t-test for the amount of change or rate of change during the observation period
Significance Level : Two-sided 5%
Indicated by : Mean and SD (standard deviation) values

4.3.2.2. Items related to scores

Items : SOFA
Analysis Group(s) : FAS and PPS
Scale : Measured value
Time Period : Observation Period
Test(s) : (Inter-group) Two-sample t-test for differences in the amount of change or rate of change during the observation period
(Intra-group) One-sample t-test for the amount of change or rate of change during the observation period

Significance Level : Two-sided 5%

Indicated by : Mean, SD (standard deviation), median and IQR (interquartile range) values

4.3.2.3. Items related to Gram staining

Item(s) : Estimation of pathogenic bacteria using Gram staining and evaluation of consistency between culture results

Analysis Group(s): FAS

Test(s) : Kappa Values

Significance Level : None

Estimations : Kappa Values

Indicated by : Number of matched cases and matching rate

4.3.3.Items Related to Patient Outcomes

4.3.3.1. Comparison of 28-day mortality between groups

Item(s) : Inter-group comparison of 28-day mortality

Analysis Group(s): FAS and PPS

Test(s) : Log-rank test

Significance Level: Two-sided 5%

Estimations : Kaplan-Meier estimates and 95% confidence intervals

Indicated by : Number of case deaths within the 28-day period, and mortality rates

4.3.3.2. Comparison of the number of 28-day ventilator free days between groups

Item(s) : Inter-group comparison of the number of 28-day ventilator free days

Analysis Group(s): FAS and PPS

Test(s) : Wilcoxon rank-sum test

Significance Level : Two-sided 5%

Estimations : Median and IQR (interquartile range) values for the number of ventilator free days

Indicated by : Median and IQR (interquartile range) values

4.4. Safety Analysis

4.4.1.Incidence Rate of Side Effects

Item(s) : Side Effects (renal dysfunction, thrombocytopenia, diarrhea, rash, convulsions)

Analysis Group(s): SAS

Test(s) : None conducted

Estimations : Incidence Rates and 95% confidence intervals

Indicated by : Number of cases that experienced side effects, and incidence rates

4.5. Subgroup Analysis

Items : Primary and Secondary Evaluation Items

Analysis Group(s): FAS

Methods : The primary and secondary evaluation items were analyzed according to the following subgroups.

For main analyses (primary evaluation items only)

1) History of antibiotic treatment from the period of initial hospitalization to assignment into a treatment group
2) At the time of assignment into a treatment group, time period spent in the ICU is 5 days or more or less than 5 days
3) Presence or absence of COPD
4) Presence or absence of head trauma
5) Post-cardiac arrest syndrome
6) Presence or absence of sepsis
7) Presence or absence of AKI (KDIGO classification Stage I or higher)
8) APACHE II of 20 or more or less than 20

For exploratory analyses (optional)

1)Geckler classification of suctioned sputum of 4 or more or less than 4
2)Classification based on Gram staining results (GPC cluster, GNR, GPC cluster + GNR)
3)Presence or absence of immunosuppression
4)Presence or absence of de-escalation
5)Classification based on sites where Gram staining was performed (Bedside or Microbiological laboratory)

4.6. Other

Other analyses were performed as needed.

5.References

1) Jumpei Yoshimura*, Kazuma Yamakawa, Takahiro Kinoshita, Satoshi Fujimi Gram stain-guided antibiotic choice: a GRACEful method to safely restrict overuse of broad-spectrum antibiotic agents: **Critical Care** 22:338. 2018

2)Vance G. Fowler Jr., Helen W. Boucher, G. Ralph Corey, *et al*: Daptomycin versus Standard Therapy for Bacteremia and Endocarditis Caused by Staphylococcus aureus: **N Engl J Med**. **355:**653-665. 2006.

6.History of Changes

Date of Change	Responsible Party	Details of Change
2018/7/11 Ver.0.1	Takeshi Morimoto	Statistical analysis plan reported in protocol paper (Trials 2018;19: 614)
2019/10/4 Ver.1.0	Takeshi Morimoto	Final version confirmed
