# Supplementary material for: Effect of Gram Stain–Guided Initial Antibiotic Therapy on Clinical Response in Patients With Ventilator-Associated Pneumonia: The GRACE-VAP Randomized Clinical Trial
Source: JAMA Netw Open. 2022 Apr 8;5(4):e226136. doi: 10.1001/jamanetworkopen.2022.6136 (PMC8994124; doi:10.1001/jamanetworkopen.2022.6136)
Supplement: Supplement 2. — eTable 1. Participating Institutions and Investigators eTable 2. Members of the Data and Safety Monitoring Board eTable 3. Antibiogram of Participating Institutions eTable 4. Microorganisms Isolated in Respiratory Samples eTable 5. Summary of the Results of Gram Staining eTable 6. Summary of Selected Antibiotic Agents eTable 7. Clinical Response of Participating Institutions eTable 8. Concordance Between Results of Gram Staining and Culture eFigure 1. Interventions eFigure 2. Twenty-Eight-Day Mortality in the Gram Stain–guided Group and Guideline-based Group [file jamanetwopen-e226136-s002.pdf]

Supplementary Online Content

Yoshimura J, Yamakawa K, Ohta Y, et al. Effect of Gram stain–guided initial antibiotic therapy on clinical response in patients with ventilator-associated pneumonia: the GRACE-VAP randomized clinical trial. *JAMA Netw Open*. 2022;5(4):e226136. doi:10.1001/jamanetworkopen.2022.6136

eTable 1. Participating Institutions and Investigators

eTable 2. Members of the Data and Safety Monitoring Board

eTable 3. Antibiogram of Participating Institutions

eTable 4. Microorganisms Isolated in Respiratory Samples

eTable 5. Summary of the Results of Gram Staining

eTable 6. Summary of Selected Antibiotic Agents

eTable 7. Clinical Response of Participating Institutions

eTable 8. Concordance Between Results of Gram Staining and Culture

eFigure 1. Interventions

eFigure 2. Twenty-Eight-Day Mortality in the Gram Stain–guided Group and Guideline-based Group

This supplementary material has been provided by the authors to give readers additional information about their work.

eTable 1. Participating Institutions and Investigators

Institution	Investigator
Chukyo Hospital	Akinori Osuka, MD, PhD
Ebina General Hospital	Takeshi Yamagiwa, MD, PhD
Hitachi General Hospital	Kensuke Nakamura, MD, PhD
Kansai Medical University Hospital	Hiroki Takahashi, MD
Kansai Medical University Medical Center	Masahiro Kawada, MD
Nagasaki University Hospital	Shuhei Yamano, MD
Osaka General Medical Center	Jumpei Yoshimura, MD
Saga University Hospital	Ryota Sakurai, MD
Sapporo City General Hospital	Akira Kodate, MD
Tajima Emergency and Critical Care Medical Center	Makoto Kobayashi, MD
University of the Ryukyus Hospital	Takayuki Taira, MD
Wakayama Medical University Hospital	Kyohei Miyamoto, MD, PhD

© 2022 Yoshimura J et al. *JAMA Network Open.*

eTable 2. Members of the Data and Safety Monitoring Board

Member name	Institution
Yuichiro Oba, MD, DTM&H	Department of General Internal Medicine and Infectious Diseases, Osaka General Medical Center
Atsushi Hiraide, MD, PhD	Department of Paramedic Science, Faculty of Health Sciences, Kyoto Tachibana University
Hitoshi Yamamura, MD, PhD	Osaka Prefectural Nakakawachi Emergency and Critical Care Center

© 2022 Yoshimura J et al. *JAMA Network Open.*

eTable 3. Antibiogram of Participating Institutions

		Methicillin-sensitive *Staphylococcus aureus*	*Pseudomonas aeruginosa*				
			PIPC	PIPC/TAZ	CAZ	CFPM	MEPM
Chukyo Hospital	2017 (%)	54	90	91	93	-	88
	2018 (%)	55	94	95	95	-	94
	2019 (%)	60	93	96	95	94	92
Ebina General Hospital	2017 (%)	66	85	85	89	94	90
	2018 (%)	68	87	87	90	93	92
	2019 (%)	57	82	82	88	89	93
Hitachi General Hospital	2017 (%)	59	90	90	90	88	94
	2018 (%)	67	81	85	85	85	89
	2019 (%)	65	88	92	91	90	91
Kansai Medical University Hospital	2017 (%)	45	91	93	94	89	92
	2018 (%)	44	97	95	96	96	95
	2019 (%)	60	92	92	92	91	92
Kansai Medical University Medical Center	2017 (%)	51	80	85	91	92	84
	2018 (%)	51	86	85	94	96	87
	2019 (%)	45	74	66	85	92	74
Nagasaki University Hospital	2017 (%)	63	84	86	85	90	88

© 2022 Yoshimura J et al. *JAMA Network Open.*

	2018 (%)	63	93	92	91	91	89
	2019 (%)	68	96	93	94	93	92
Osaka General Medical Center	2017 (%)	57	72	74	77	82	80
	2018 (%)	68	77	77	79	83	85
	2019 (%)	60	90	93	92	94	95
Saga University Hospital	2017 (%)	58	96	97	95	96	99
	2018 (%)	58	96	96	94	95	100
	2019 (%)	56	93	95	91	94	97
Sapporo City General Hospital	2017 (%)	-	-	-	-	-	-
	2018 (%)	77	87	88	95	96	88
	2019 (%)	81	95	95	97	94	95
Tajima Emergency and Critical Care Medical Center	2017 (%)	58	97	99	98	97	95
	2018 (%)	69	94	94	94	93	94
	2019 (%)	61	97	97	96	97	96
University of the Ryukyus Hospital	2017 (%)	45	68	69	84	83	76
	2018 (%)	53	84	86	90	93	88
	2019 (%)	55	81	85	87	90	89
Wakayama Medical University Hospital	2017 (%)	70	91	93	93	91	92
	2018 (%)	73	91	93	92	91	88
	2019 (%)	73	90	92	91	91	92

Abbreviations: CAZ, ceftazidime; CFPM, cefepime; MEPM, meropenem; PIPC, piperacillin; PIPC/TAZ, piperacillin/tazobactam.

© 2022 Yoshimura J et al. *JAMA Network Open.*

eTable 4. Microorganisms Isolated in Respiratory Samples

	**Gram stain-guided group (n=103)**	**Guideline-based group (n=103)**
Gram-positive bacteria		
Staphylococcus aureus	50	53
MRSA	9	13
Streptococcus pneumonia	1	6
Corynebacterium spp.	3	1
Other streptococci	54	53
Other Gram-positive bacilli	1	1
Gram-negative bacteria		
Klebsiella spp.	18	16
Haemophilus influenza	9	11
Enterobacter spp.	10	8
Escherichia coli	7	9
Pseudomonas aeruginosa	2	9
Serratia marcescens	6	2
Moraxella catarrhalis	6	1
Acinetobacter baumannii	5	1
Citrobacter spp.	1	2
ESBL-producing Enterobacteriaceae	4	2
Other Gram-negative bacteria	5	7
MDR Gram-negative bacteria	0	0

Abbreviations: ESBL, extended-spectrum beta-lactamase; MDR, multi-drug resistant; MRSA, methicillin-resistant *Staphylococcus aureus*.

© 2022 Yoshimura J et al. *JAMA Network Open.*

eTable 5. Summary of the Results of Gram Staining

	Gram stain-guided group (n=103)	Guideline-based group (n=103)
Gram-positive cocci chains	52	50
Gram-positive cocci clusters	56	55
Gram-positive bacilli	28	28
Gram-negative rods	65	57
Absence of any microorganisms	5	9

© 2022 Yoshimura J et al. *JAMA Network Open.*

eTable 6. Summary of Selected Antibiotic Agents

	**Gram stain-guided group (n=103)**	**Guideline-based group (n=103)**
Anti-MRSA agent and anti-pseudomonal agent	43	103
Anti-MRSA agent and non-pseudomonal β-lactam	1	0
Anti-MRSA agent	19	0
Anti-pseudomonal agent	29	0
Non-pseudomonalβ-lactam	11	0

Abbreviation: MRSA, methicillin-resistant *Staphylococcus aureus*.

© 2022 Yoshimura J et al. *JAMA Network Open.*

eTable 7. Clinical Response of Participating Institutions

Participating Institutions, n (%)	Clinical Response		Risk difference (95% CI)
	Gram stain-guided group	Guideline-based group	
Osaka General Medical Center	36/40 (80.0)	38/41 (92.7)	-0.027 (-0.15-0.10)
Hitachi General Medical Hospital	24/37 (64.9)	20/37 (54.1)	0.11 (-0.11-0.33)
Kansai Medical University Medical Center	5/9 (55.6)	5/8 (62.5)	-0.069 (-0.54-0.40)
Kansai Medical University Hospital	5/5 (100)	1/4 (25.0)	0.75 (0.33-1.17)
Ebina General Hospital	2/2 (100)	4/5 (80.0)	0.20 (-0.15-0.55)
Sapporo City General Hospital	1/2 (50.0)	4/4 (100)	-0.5 (-1.19-0.19)
Wakayama Medical University Hospital	3/4 (75.0)	1/1 (100)	-0.25 (-0.67-017)
Chukyo Hospital	2/2 (100)	1/1 (100)	-
Nagasaki University Hospital	0/1 (0)	0/0	-
University of the Ryukyus Hospital	0/0	0/1 (0)	-
Saga University Hospital	1/1 (100)	0/0	-
Tajima Emergency and Critical Care Medical Center	0/0	0/1 (0)	-

© 2022 Yoshimura J et al. *JAMA Network Open.*

eTable 8. Concordance Between Results of Gram Staining and Culture

**GPC cluster**				
		Staphylococcus aureus growth in culture[a]		
		Yes	No	Total
GPC cluster in Gram staining	Yes	86	25	111
	No	17	78	95
	Total	103	103	206
**GPC chain**				
		Gram-positive Streptococcus species in culture[b]		
		Yes	No	Total
GPC chain in Gram staining	Yes	79	23	102
	No	35	69	104
	Total	114	92	206
**GPB**				
		Gram-positive bacilli in culture[c]		
		Yes	No	Total
GPB in Gram staining	Yes	4	52	56
	No	1	149	150
	Total	5	201	206
**GNR**				
		Gram-negative pathogen growth in culture[d]		
		Yes	No	Total
GNR in Gram staining	Yes	78	44	122
	No	16	68	84
	Total	94	112	206

Abbreviations: GPB, Gram-positive bacilli; GPC, Gram-positive cocci; GNR, Gram-negative rods.

[a] Sensitivity of GPC cluster in Gram staining: 86/103=83·5%; specificity of GPC cluster in Gram staining: 78/103=75·7%.

[b] Sensitivity of GPC chain in Gram staining: 79/114=69·3%; specificity of GPC chain in Gram staining: 69/92=67·4%.

[c] Sensitivity of GPB in Gram staining: 4/5=80·0%; specificity of GPB in Gram staining: 149/201=74·1%.

[d] Sensitivity of GNR in Gram staining: 78/94=83·0%; specificity of GNR in Gram staining: 68/112=60·7%.

© 2022 Yoshimura J et al. *JAMA Network Open.*

eFigure 1. Interventions

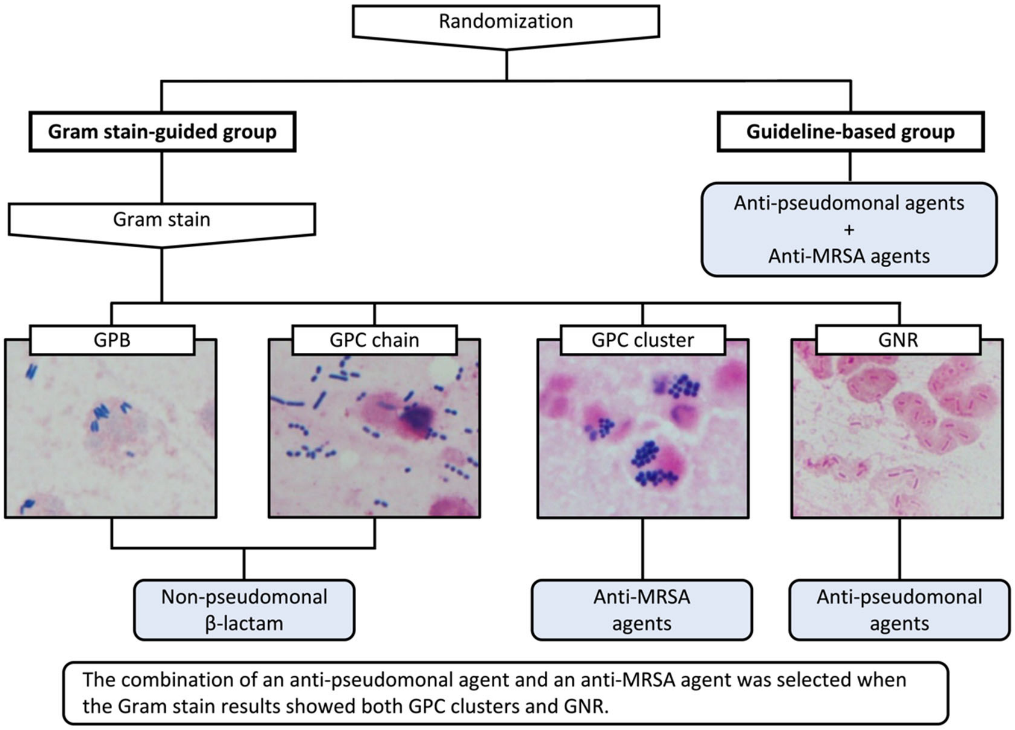

Abbreviations: GPB, Gram-positive bacilli; GPC, Gram-positive cocci; GNR, Gram-negative rods; MRSA, methicillin-resistant *Staphylococcus aureus*.

© 2022 Yoshimura J et al. *JAMA Network Open.*

eFigure 2. Twenty-Eight-Day Mortality in the Gram Stain–guided Group and Guideline-based Group

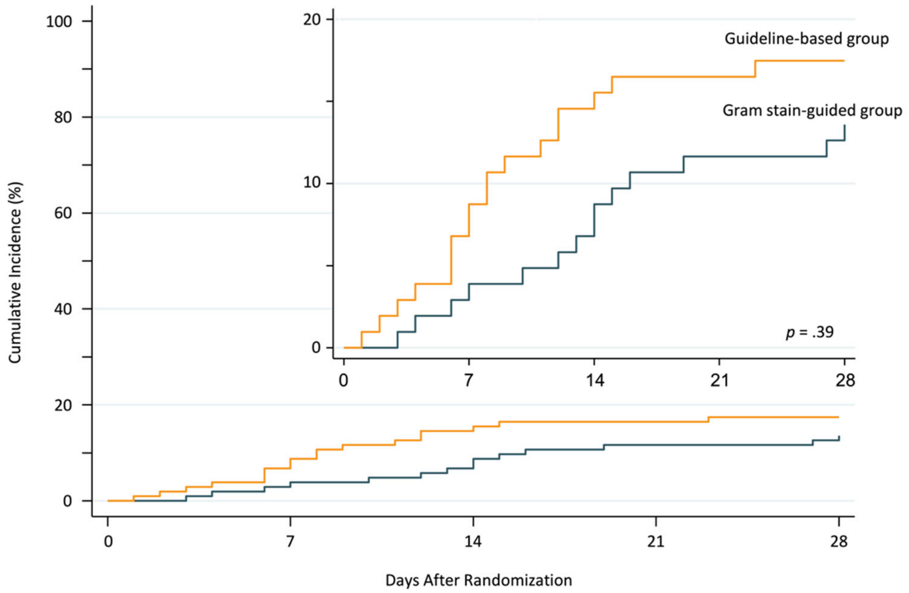

**Guideline-based group**					
Number at risk	103	94	87	86	85
Cumulative number of events		9	16	17	18
Cumulative incidence		8.7%	15.5%	16.5%	17.5%
**Gram stain-guided group**					
Number at risk	103	99	94	91	89
Cumulative number of events		4	9	12	14
Cumulative incidence		3.9%	8.7%	11.7%	13.6%

The inset shows the same data on an expanded y axis.

© 2022 Yoshimura J et al. *JAMA Network Open.*
