# Supplementary material for: Effect of Gram Stain–Guided Initial Antibiotic Therapy on Clinical Response in Patients With Ventilator-Associated Pneumonia: The GRACE-VAP Randomized Clinical Trial
Source: JAMA Netw Open. 2022 Apr 8;5(4):e226136. doi: 10.1001/jamanetworkopen.2022.6136 (PMC8994124; doi:10.1001/jamanetworkopen.2022.6136)
Supplement: Supplement 3. — Data Sharing Statement [file jamanetwopen-e226136-s003.pdf]

Data Sharing Statement

Yoshimura. Effect of Gram Stain-Guided Initial Antibiotic Therapy on Clinical Response in Patients With Ventilator-Associated Pneumonia. *JAMA Netw Open*. Published April 08, 2022. doi:10.1001/jamanetworkopen.2022.6136

Data
Data available: Yes
Data types: Deidentified participant data
How to access data: How to access data: Corresponding author email: kyamakawa-osk@umin.ac.jp
When available: beginning date: 04-01-2024

Supporting Documents
Document types: None

Additional Information
Who can access the data: Researchers whose proposed use of the data has been approved
Types of analyses: For a specified purpose
Mechanisms of data availability: After approval of a proposal
